# Insights into the microbiome assembly during different growth stages and storage of strawberry plants

**DOI:** 10.1186/s40793-022-00415-3

**Published:** 2022-04-28

**Authors:** Expedito Olimi, Peter Kusstatscher, Wisnu Adi Wicaksono, Ahmed Abdelfattah, Tomislav Cernava, Gabriele Berg

**Affiliations:** 1 Institute of Environmental Biotechnology, Graz University of Technology, Graz, Austria; 2 Leibniz Institute for Agricultural Engineering and Bioeconomy (ATB), Potsdam, Germany; 3 Institute for Biochemistry and Biology, University of Potsdam, Potsdam, Germany

**Keywords:** *Fragaria* × *ananassa*, Microbiome assembly, Fruit pathogens, Bacterial communities, Fungal communities, Amplicon sequencing, CLSM

## Abstract

**Background:**

Microbiome assembly was identified as an important factor for plant growth and health, but this process is largely unknown, especially for the fruit microbiome. Therefore, we analyzed strawberry plants of two cultivars by focusing on microbiome tracking during the different growth stages and storage using amplicon sequencing, qPCR, and microscopic approaches.

**Results:**

Strawberry plants carried a highly diverse microbiome, therein the bacterial families *Sphingomonadaceae* (25%), *Pseudomonadaceae* (17%), and *Burkholderiaceae* (11%); and the fungal family *Mycosphaerella* (45%) were most abundant. All compartments were colonized by high number of bacteria and fungi (10^7^–10^10^ marker gene copies per g fresh weight), and were characterized by high microbial diversity (6049 and 1501 ASVs); both were higher for the belowground samples than in the phyllosphere. Compartment type was the main driver of microbial diversity, structure, and abundance (bacterial: 45%; fungal: 61%) when compared to the cultivar (1.6%; 2.2%). Microbiome assembly was strongly divided for belowground habitats and the phyllosphere; only a low proportion of the microbiome was transferred from soil via the rhizosphere to the phyllosphere. During fruit development, we observed the highest rates of microbial transfer from leaves and flowers to ripe fruits, where most of the bacteria occured inside the pulp. In postharvest fruits, microbial diversity decreased while the overall abundance increased. Developing postharvest decay caused by *Botrytis cinerea* decreased the diversity as well, and induced a reduction of potentially beneficial taxa.

**Conclusion:**

Our findings provide insights into microbiome assembly in strawberry plants and highlight the importance of microbe transfer during fruit development and storage with potential implications for food health and safety.

**Supplementary Information:**

The online version contains supplementary material available at 10.1186/s40793-022-00415-3.

## Background

The plant microbiome consists of bacteria, archaea, fungi, viruses, microalgae, oomycetes and small protozoans [1, 2]. Microorganisms occur specifically in various plant compartments, which are mainly divided into belowground habitats (e.g. roots and their rhizosphere) and aboveground habitats (e.g. leaves, flowers, fruits, and stem), and are transferred to the next generation by propagules including seeds [3, 4]. Previous studies have indicated that plant compartments are connected yet distinct in their microbiome composition [5, 6], even at strain level [7]. Significant variation in the microbiome between the aboveground and belowground compartments is commonly observed, which can be explained by contrasting biotic and abiotic factors [8, 9]. The plant microbiome contributes to the plant phenotype [10, 11], can influence plant metabolism [12, 13], and plays important roles in plant health and productivity [14–16]. Therefore, the plant and its associated microbiome have collectively been referred to as the plant holobiont [17]. While belowground plant microbiome assembly, e.g. in the rhizosphere, is well-studied, less is known about connections to the phyllosphere and developing fruits [10].

Strawberry (*Fragaria x ananassa* [Weston] Rozier), which belongs to the *Rosaceae* plant family, is a high-value horticultural crop with significant nutritional benefits [18]. Special attributes such as its red color, juicy texture, and sweet fruity flavor have made strawberries one of the most desired fruits [19, 20]. More than 300 compounds including volatiles are involved in its unique flavor to which metabolic interplay with bacteria contributes [12, 21]. In addition, remarkable health benefits, attributed to strawberry anthocyanins, were revealed by studies involving animal models and human clinical trials [22–25]. Owing to the abovementioned benefits, the global production reached 14.6 million tonnes in 2019 [26]. However, strawberry production and storage are challenged by a plethora of constraints caused by fungal and bacterial pathogens, as well as nematodes, both in open and closed farming systems [27, 28]. The use of conventional crop protection strategies is increasingly limited due to phased-out chemicals that are linked to environmental and human health concerns [29]. Therefore, the search for alternative strategies utilizing the plant’s microbiome has driven the development of antagonistic and plant growth-promoting bioinoculants [30, 31]. In addition, fruit-associated microbes were previously discussed to influence their shelf life [32, 33]. Different studies revealed a plant species-specific, strawberry-associated microbiome [34, 35], influenced by management and linked with the phyllosphere and rhizosphere plant-soil feedback [36, 37]. Using targeted approaches to induce microbiome changes in plants, and thus also in strawberry fruit, could not only provide a potential strategy for improving pre- and postharvest plant healht, but also food quality and safety.

This study aimed at investigating the bacterial and fungal microbiome assembly in different plant compartments (leaves, flowers, fruits, rhizosphere, soil) and cultivars ('Mara des Bois', 'White 90 ananas') during fruit development of strawberries. In addition, we focused on microbiome changes that are evoked by storage and disease of fruits. Specifically, we studied (I) how the microbial community composition and structure vary among plant compartments and cultivars, (II) which proportion of the microbiome is shared in the phyllosphere and potentially transferred during fruit development, and (III) how postharvest cold storage and disease incidence induce changes in the fruit microbiome.

## Methods

### Experimental design and sampling

Plants of two strawberry cultivars, *Fragaria x ananassa* ‘Mara des Bois’ (henceforth referred as cultivar 1) and ‘White ananas’ (cultivar 2), were bought from a plant nursery and planted at Graz University Botanical Garden (47.081 N, 15.4549E), and sampled between spring (April 2020) to late summer (August 2020). Twenty strawberry plants per cultivar were grown and no fertilizer or plant protectants were used, except the physical removal of weeds. ‘Mara des Bois’ is a hybrid from a cross of four European garden-type strawberries (Hami Gento × Ostara) × (Red Gauntlet × Korona) [38]. It is an everbearing, highly productive cultivar and much desired on the European fruit market [39]; strawberry plants of this cultivar are tolerant to powdery mildew (caused by *Podosphaera aphanis*). On the other hand, in contrast to the usual red pigmentation of strawberry, ‘White ananas’ is characterized by a white coloration of ripe fruits. This has been attributed to the deficiency of Fra a 1-A proteins, which are critical in pigment biosynthesis [40, 41]. The soil was described as loamy and had a pH of 7.55. Soil mineral content and other physico-chemical properties are highlighted in Additional File 1: Table S1.

Two months after planting, samples including bulk soil, rhizosphere (roots with attached soil), leaves, and flowers (anthosphere) were collected. From the whole field, bulk soil samples (n = 6) were taken from six random locations at a depth of approximately 5–10 cm between rows free from plant influence to represent the natural soil from this field. Subsequently, to obtain a sufficient amount of material, for each cultivar and compartment (rhizosphere, leaves and flowers), six biological replicates, with each replicate composed of samples from at least two adjacent plants, were collected. Rhizosphere sampling involved partially digging out actively growing roots and detaching the roots from the plant, followed by light shaking of the roots to remove loosely attached soil. For leaf sampling, a total of six trifoliate strawberry leaves from two adjacent plants (three random leaves per plant) were pooled to constitute a single biological replicate; similarly, a total of six flower samples (including stamen, pistil, and flower stalk; three random flowers per plant) were pooled and treated as a single biological replicate. Subsequently, sampling of the immature and ripe fruits (carposphere) was performed as follows. Immature fruits were collected one week after flower opening, while ripe fruits were picked at the full ripening stage. One part of the ripe fruits (six samples of each cultivar) was stored at 4 °C for one week after harvest before further processing. To investigate fruit microbiome changes induced due to disease, we sampled diseased fruits from plants of each cultivar; these samples showed visible signs of gray mold rot disease caused by fungi within the genus *Botrytis* (especially *B. cinerea*). Sampling was carefully implemented to avoid contamination by disinfecting utilized tools with Bacillol (PAUL HARTMANN GmbH, Wiener Neudorf, Austria), and changing or disinfecting hand gloves between handling of different samples. All samples were placed in sterile bags and temporarily stored in a cooling box before being transferred to our laboratory facility within two hours for subsequent processing and storage.

### Sample processing and DNA extraction

Soil samples were transferred to 2-mL tubes and stored at −20 °C. Processing of the rhizosphere, leaves, flowers, and immature fruits involved grinding the samples in a mortar and adding 4 mL sodium chloride buffer (0.85%). Thereafter, 2 mL of the homogenate were centrifuged at 16,000 g for 15 min at 4 °C, and the obtained pellet was stored at −20 °C prior to DNA extraction. For processing ripe fruits, 4 mL sodium chloride buffer and samples were gently squeezed in stomacher bags followed by centrifugation of the homogenate and pelleting the sample as described above. Total DNA extraction was performed using FastDNA™ SPIN Kit for Soil (MP Biomedicals; United States) following the manufacturer’s instructions. DNA was quality checked using a Nanodrop 2000 (Thermo Scientific, Wilmington, DE, USA) and stored at -20 °C until PCRs were carried out.

### Quantification of the total bacterial and fungal community using real-time quantitative PCR (qPCR)

For determining gene copy numbers of bacteria and fungi within samples, qPCR was performed using the primer pairs Unibac-II-515f/Unibac-II-806r for bacteria (10 μM each; [42]) and ITS1f/ITS2r for fungi (10 μM each; [43]). Reactions were performed in a total volume of 10 μL in a reaction mix composed of 5 μL of KAPA SYBR Green (Bio-Rad, Hercules, CA, U.S.A.), 0.5 μL of each primer, 3 μL of PCR grade water and 1 μL template DNA (samples were diluted 1:10 in PCR grade water). For each sample, amplifications were conducted in triplicates using a Rotor-Gene™ 6000 series (Corbett Research, Sydney, Australia) thermal cycler with the following program settings: initial denaturation (95 °C,5 min) followed by 35 cycles of denaturation (95 °C,10 s); annealing (54 °C, 15 s); extension (72 °C, 10 s); then melt down from 72 to 96 °C. Serial dilutions of standards containing defined copy numbers were generated according to [42] and used for the calculation of gene copy numbers in different samples. For bacterial standards a DNA template of *Bacillus* sp., while *Penicillium* sp. was used for ITS standards. A standard regression curve was used to fit the gene copy numbers of the analyzed samples and the numbers per gram of the samples computed.

### Amplicon library preparation

Extracted DNA was used for Illumina library preparation that was based on amplicons of the hypervariable V4 region of the bacterial 16S rRNA gene and the ITS1 regions of fungal DNA. For the bacterial community analysis, we employed one-step PCR using the primer pair 515F (5’-CTTGGTCATTTAGAGGAAGTAA-3’) and 806R (5’-GCTGCGTTCTTCATCGATGC-3’) for amplicon library preparation [44–46]. Both forward and reverse primers contained sample specific barcodes, facilitating multiplexed sequencing.

One microliter (µL) of extracted DNA was used in each 30-µL reaction. The reaction mixture contained 6 µL (5xTaq &GO, PCR pre-mix, MP Biomedicals), 0.6 µL (10 µM 515F/806R) primers, 0.45 µL (50 µM mPNA and pPNA), and 20.9 µL of PCR grade water. The peptide nucleic acid (PNA) PCR clamps were used to block the amplification of plastid and mitochondrial 16S rRNA gene of plants during the PCR amplification of bacterial community [47, 48]. All reactions were performed in triplicates on a thermocycler (Bio-metra GmbH, Jena, Germany). The PCR program included an initial denaturation (96 °C, 5 min), followed by 30 cycles (94 °C for 60 s, 78 °C PNA step for 5 s, 54 °C for 1 min, 74 °C for 60 s), followed by 74 °C for 10 min and then cooled down to 10 °C.

For the library preparation of the fungal community we used the primer pair ITS1f (5’-CTTGGTCATTTAGAGGAAGTAA-3’) and ITS2r (5’-GCTGCGTTCTTCATCGATGC-3’) [43, 49]. The preparation followed a two-step amplification approach involving amplification of the ITS1/ITS2 region and subsequent attachment of sample specific barcodes. All amplifications were performed in triplicates. In the first PCR, 1 µL of DNA template was used for each 10 µL reaction; the reaction mixture contained 2 µL (5 × Taq & Go), 1.2 µL (25 mM MgCl_2_), 0.1 µL (10 µM ITS1/ITS2 primers with pads), and 5.6 µL of PCR grade water. The second amplification was performed using 2 µL of the first PCR product in 30 µL reaction mixture. Each reaction mixture was composed of 6 µL (5xTaq &GO), 1.2 µL (10 µM; Forward/Reverse barcode primers), and 19.6 µL of PCR grade water. The PCR program for the first amplification step included an initial denaturation (96 °C, 5 min), followed by 35 cycles (95 °C for 30 s, 58 °C for 35 s, 72 °C for 40 s), followed by 72 °C for 10 min and then cool down to 10 °C. The subsequent reaction program involved: initial denaturation (95 °C, 5 min), then 15 cycles (95 °C for 30 s, 53 °C for 30 s, 72 °C for 30 s), followed by 5 min at 72 °C and cooling to 10 °C. Successful PCR amplifications at the correct amplicon size were confirmed by gel electrophoresis. PCR amplicons were purified using the Wizard SV Gel and PCR Clean-Up System (Promega, Madison, WI) following the manufacturer’s instructions. Purified PCR amplicons were quantified using a Nanodrop 2000 (Thermo Scientific, Wilmington, DE, USA) and pooled in equimolar concentrations. Paired-end Illumina MiSeq 2 × 300 sequencing of the amplicon library was performed by the sequencing provider GENEWIZ (Berlin, Germany). All raw reads obtained from the sequencing company were deposited at the European Nucleotide Archive (ENA) under study accession number PRJEB47432.

### Bioinformatic pipeline

Paired-end reads were quality-checked and demultiplexed using cutadapt [50]. Demultiplexed reads were further analysed using the open-source QIIME2 version 2020.6.0 pipeline (https://qiime2.org) [51]. Primer sequences were removed and DADA2 algorithm in QIIME2 was employed to quality filter, denoise and remove chimeric sequences [52], thus generating representative sequences, known as amplicon sequence variants (ASVs), and a feature table. Taxonomic assignments were performed using the reference databases SILVA132 for 16S rRNA gene fragments [53, 54] and UNITE v7 [55] for fungal sequence reads.

### Statistical analysis

The R version 4.0.3 [56] was used for performing statistical analysis and visualization; supplemented by Microbiome analyst [57]. Kruskal–Wallis followed by the Wilcoxon-Mann–Whitney test was performed to detect significant differences in microbial gene copies between different plant compartments and cultivars. For microbiome analyses, the obtained microbial ASV tables and taxonomic classifications were uploaded into R via phyloseq [58] and vegan [59] packages. Both alpha and beta diversity analyses were performed on datasets rarefied to minimum sampling depths of 1000 and 2500 reads per sample for bacterial and fungal communities respectively as shown in Additional file 1: Fig. S1. To illustrate sequencing depth, alpha rarefaction curves were plotted using ranacapa Shinny web application [60]. Microbial alpha diversity was determined using Shannon diversity and richness (Observed ASVs) indices. For bacterial community, parametric analysis involving two-way ANOVA, followed by post-hoc analysis using TUKEY-HSD test correction was employed to determine the differences in α-diversity between compartments and cultivars. Meanwhile, due to non-parametric characteristics of the fungal dataset, we employed the non-parametric Kruskal–Wallis test, followed by the Wilcoxon-Mann–Whitney test, and Benjamini-Hochberg’s FDR method for diversity comparisons between compartments and cultivar groups. Beta diversity analyses based on normalized Bray–Curtis dissimilarity matrix was performed using permutational analysis of variance (PERMANOVA, 999 permutations) to reveal the compartment type and cultivar effects on microbial community composition. The distance matrices were visualized using principal coordinates analysis (PCoA). Stacked bar plots were used for visualization of microbial taxonomic composition. Additionally, Linear discriminant analysis Effect Size (LEfSE) [61] was implemented to identify the taxa which explained the differences between strawberry compartments, cultivars, as well as fruit development stages. We used the SourceTracker2 package in R [62] to estimate the proportion of microbiome transferred among strawberry compartments. This tool is designated to estimate the proportion of microbiome that originates from a set of source-sink environments. We tested each strawberry compartment for being a source or a sink in a similar way as it was already described [63].

### Fluorescent in situ hybridization and confocal laser scanning microscopy (FISH-CLSM)

We observed the native colonization patterns of the strawberry fruit microbiota by FISH-CLSM, using a Leica TCSSPE confocal scanning microscope (Leica Microsystems, Mannheim, Germany) with the oil immersion objective lens Leica ACS APO 40 × oil CS. Briefly, strawberry fruit tissues (including a thin fruit peeling and fruit fresh) were fixed with 4% paraformaldehyde/phosphate-buffered saline overnight at 4 °C prior to application of the FISH protocol according to [64]. Precisely, the Cy3-labelled EUB338MIX [65, 66] was used to stain overall bacteria colonization; for specific visualization of *Firmicutes* the Cy5-labeled LGC-mix was used [67], and ALEXA488-labeled GAM42a for *Gammaproteobacteria* [68]. For contrasting host cell walls, FISH samples were treated with calcofluor White [69]. Micrographs visualizing the bacterial colonization were generated by maximum projections of optical z-stacks slices.

## Results

### Abundances of bacteria and fungi in different strawberry compartments

**Fig. 1:**
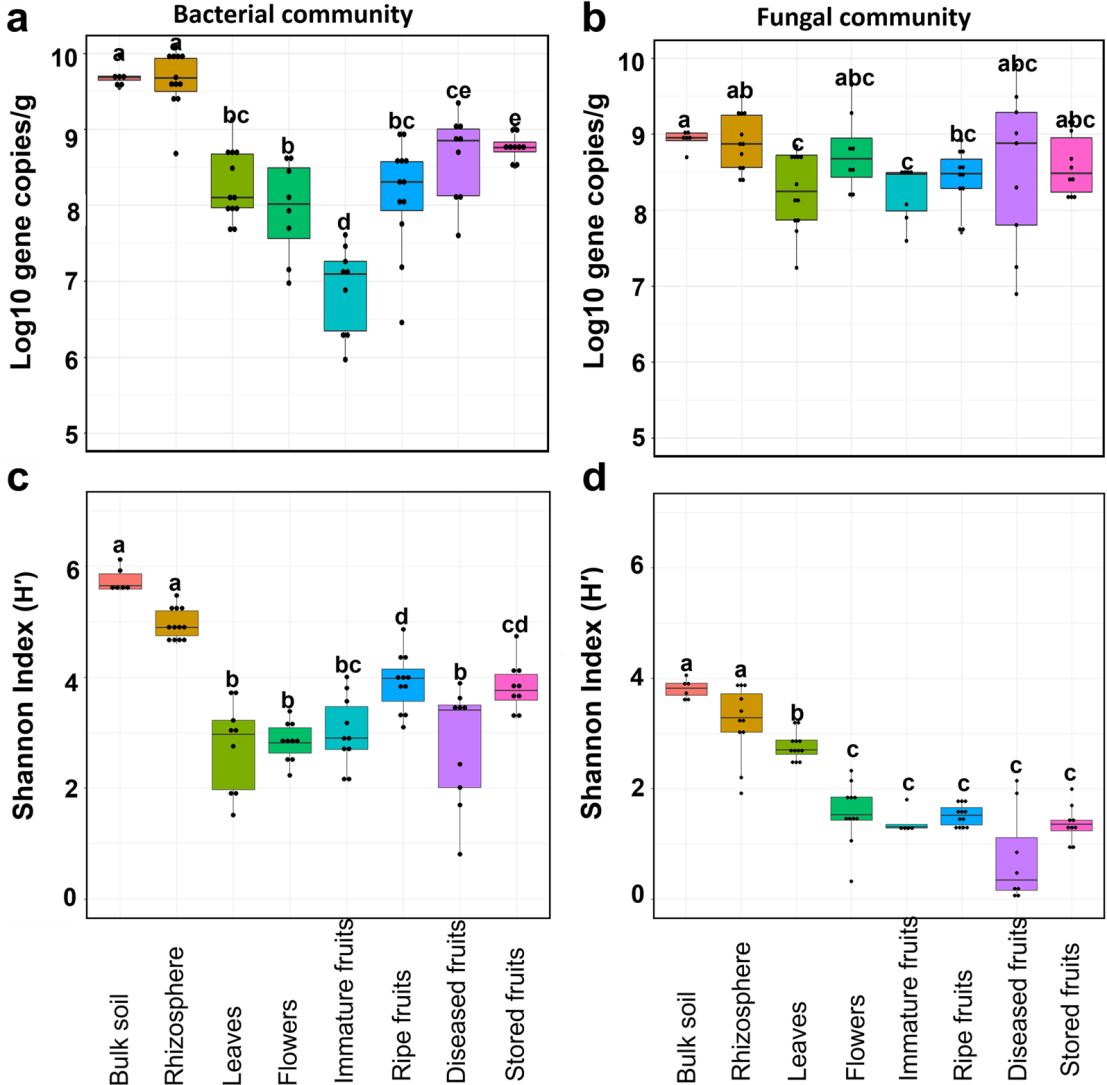
Bacterial and fungal community abundances and alpha diversity (Shannon index). Boxplots indicate observed bacterial (**a**) and fungal (**b**) gene copy numbers as well as bacterial (**c**) and fungal (**d**) diversity in soil, rhizosphere, and strawberry phyllosphere compartments. Significant differences (*P* < 0.05) were respectively obtained by pairwise Wilcoxon Rank Sum Test (for abundance) and TUKEY-HSD test (alpha diversity), and are indicated by different letters above the boxplots

Gene copy numbers of bacterial 16S rRNA fragments and the fungal ITS region per gram fresh weight of each strawberry compartment (leaves, flowers, fruits, rhizosphere, and soil) were quantified and ranged between 2.83 × 10^7^ and 1.17 × 10^10^, and between 4.27 × 10^8^ and 3.38 × 10^9^ respectively. There was a significant effect of compartment type (Kruskal–Wallis: *P* < 0.005) on bacterial and fungal gene copy numbers (Fig. 1a, b). Moreover, significant differences (*P* < 0.05) in microbial abundance between belowground (rhizosphere and bulk soil) and aboveground (leaves, flowers, immature fruits, and ripe fruits) compartments were observed (Additional file 1: Table S2). Bacterial abundance was high in the rhizosphere (mean: 1.17 × 10^10^) and soil (1.07 × 10^10^) but not significantly different. Interestingly, bacterial abundance among the phyllosphere compartments decreased from leaves (6.3 × 10^8^) to flowers (3.51 × 10^8^) and immature fruits (2.83 × 10^7^), but then increased in ripe fruits (5.98 × 10^8^). Diseased fruits (1.5 × 10^9^) and stored fruits (1.25 × 10^9^) contained more bacteria as compared to ripe fruits at harvest. Meanwhile, the fungal abundance in soil and the rhizosphere was significantly higher in comparison to leaves and immature fruits (Fig. 1b); however, no significant differences were observed among the phyllosphere compartments (Additional file 1: Table S4). As with the bacterial community, the fungal abundance was higher in diseased (3.38 × 10^9^) and stored (1.16 × 10^9^) fruits in comparison to ripe fruits (6.98 × 10^8^), but not significantly different. The fungal abundance in cultivar 1 was significantly higher than in cultivar 2 (Kruskal–Wallis test: P = 0.002); while no significant difference (Kruskal–Wallis test: *P* = 0.496) in bacterial abundance was observed between the cultivars. To estimate the number of ingested microorganisms upon consumption of one ripe strawberry fruit, we multiplied the calculated microbial (16S rRNA and ITS) gene copy numbers per gram of ripe fruit with the estimated mean weight of a standard strawberry fruit (16 g). The amount of microbiota consumed was calculated as the ratio of total gene copies and the average 16S rRNA (4.2) and ITS (113) gene copies per genome as earlier estimated [70, 71]. Thus, we found that consuming a strawberry fruit results in the ingestion of 2.28 × 10^9^ and of 9.88 × 10^7^ bacterial and fungal cells respectively.

### Compartment type defines diversity of the strawberry microbiome

Amplicon sequencing of bacterial and fungal communities respectively yielded 2,309,124 and 1,383,225 high-quality reads. After filtering to remove non-target reads (e.g. chloroplast or mitochondria), sequences were assigned to 6049 bacterial and 1501 fungal amplicon sequence variants (ASVs). With respect to compartment type, one-way ANOVA and Kruskal–Wallis tests that were employed respectively on bacterial and fungal communities revealed significant differences (*P* < 0.001) in microbial alpha diversity between the phyllosphere and belowground compartments. The average alpha diversity based on the Shannon index was generally higher in belowground compared to the phyllosphere compartments (bacteria: H´ = 5.23 vs 3.2, and fungi: H´ = 3.42 vs 1.64) (Fig. 1c, d). There were no significant variations in alpha diversity between soil and rhizosphere for either community (*P* > 0.05), although the soil diversity was higher (bacteria: H' = 5.75; fungi: H' = 3.82) in comparison to rhizosphere (bacteria: H' = 4.97; fungi: H' = 3.21). Furthermore, no significant difference in bacterial diversity was observed between ripe- and stored fruits; however, diseased fruits were significantly less diverse in comparison to either ripe or stored fruits. Regarding the fungal community, the diversity was significantly higher in leaves (H´ = 2.77) than in flowers, immature fruits, and ripe fruits; however, the fungal diversity in ripe and stored fruits was higher than in diseased fruits, but not statistically different (Fig. 1d). Generally, we observed a significant influence of the cultivar (Kruskal–Wallis: P < 0.05) on microbial diversity, but compartment-specific differences were only noticeable in leaves for the bacterial community (Additional File 1: Tables S3 and S4). In addition, as shown in Additional File 1: Fig. S2, microbial richness (observed ASVs) showed a similar trend to microbial diversity, albeit with slight variations; however, chao1 which estimates overall community richness was slightly higher (Additional File 1: Fig.S2c and d) in comparison to observed ASVs (Additional File 1: Fig.S2a and b).

### Microbial composition and structure varied between the phyllosphere and belowground communities

**Fig. 2:**
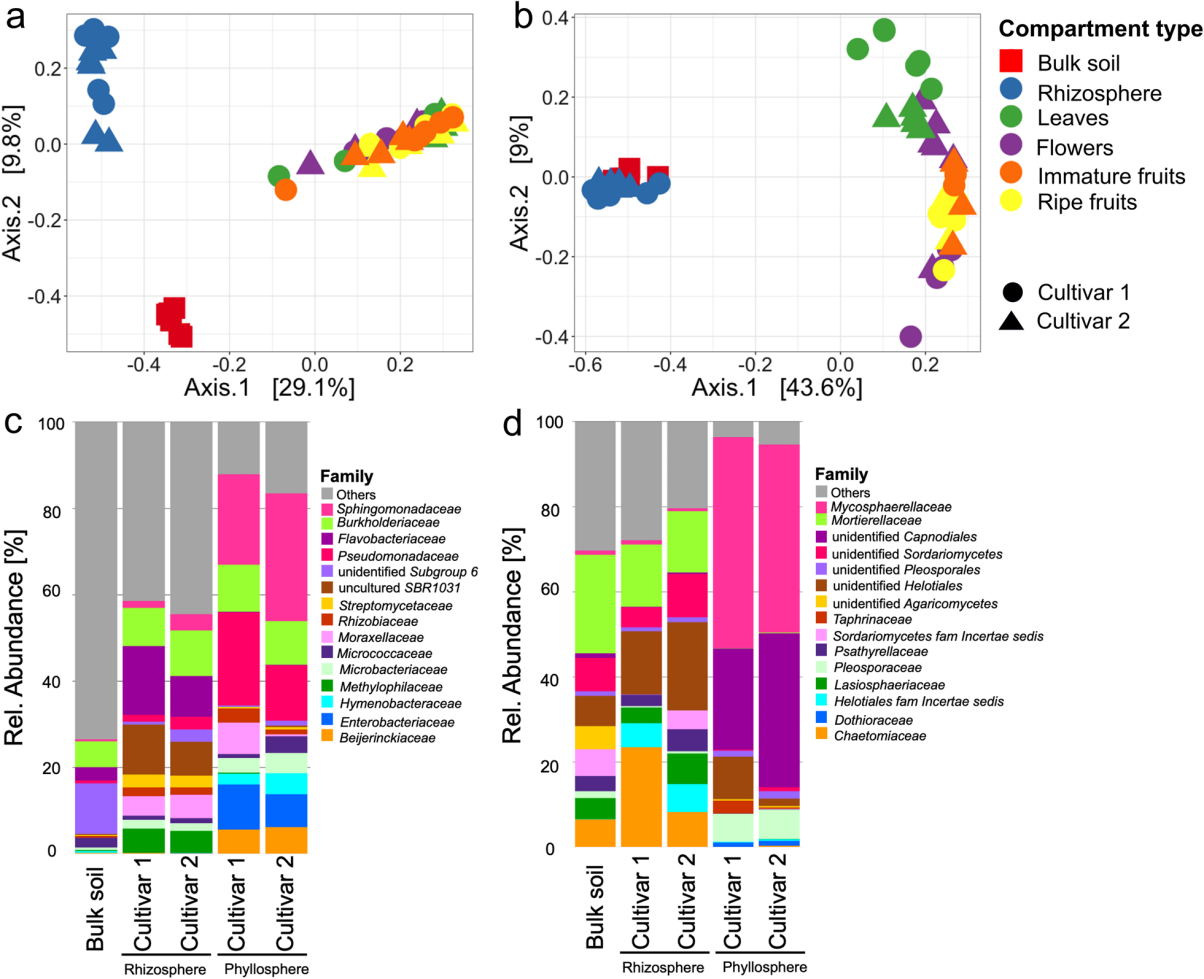
Principle Coordinates Analysis (PCoA) and stacked bar plot representation of plant-associated microbial communities with respect to above-and belowground compartments in the different strawberry cultivars. Bacterial (**a** and **c**) and fungal (**b** and **d**) community clustering and composition in the rhizosphere, soil, and phyllosphere compartments (average of leaves, flowers, and fruits). The stacked bar plots are based on the top 15 families, while all remaining taxa were included in “Others”. Colors represent the compartment type, while shapes show soil and strawberry cultivar

Permutational multivariate analyses of variance (PERMANOVA) of Bray–Curtis distance matrix revealed that compartments (R2; bacteria:0.451 and fungi: 0.609) significantly contributed to microbial community differences (*P* = 0.001). Visualization of Bray–Curtis distance matrices by Principal Coordinates Analysis (PCoA) further showed compartment-specific community separation (bacterial: *P* = 0.001, R2 = 0.199; fungal community: *P* = 0.001, R2 = 0.304). Moreover, a separation between strawberry cultivars among belowground and aboveground compartments was visible (Fig. 2a, b).

The taxonomic assignment of ASVs in the phyllosphere and belowground compartments revealed 31 different bacterial phyla, and 394 families; while fungal ASVs were assigned to 6 phyla, and 145 families. Among the bacterial phyla, *Proteobacteria* were predominant with an average abundance of 50.1%, followed by *Bacteroidetes* (12.3%), *Actinobacteria* (11.2%), *Acidobacteria* (8.3%), *Chloroflexi* (7.4%), and *Firmicutes* (2.8%). The fungal phyla were predominated by *Ascomycota* (76%), *Mortierellomycota* (11.3%), and *Basidiomycota* (11.1%); as well *Chytridiomycota* and *Mucoromycota*. To visualize the phyllosphere and belowground microbial composition, the top 15 families were shown, while the remaining taxa were classified as others (Fig. 2c, d). At family level, a high proportion of taxa were classified as “others” (46.2% average abundance) and were respectively high in soil (74.6%) and the rhizosphere (49.6%) as compared to phyllosphere compartments (14.3%); this reflected the higher diversity in soil and rhizosphere compared with phyllosphere compartments. The most abundant bacterial family was *Sphingomonadaceae* (average abundance: 10.3%), which was highly present in phyllosphere compartments, followed by *Burkholderiaceae* (9.4%), *Flavobacteriaceae* (5*.5%*), and *Pseudomonadaceae* (5.1%) (Fig. 2c). Bacterial families including *Enterobacteriacea*e, *Bacillaceae*, *Hymenobacteraceae* and *Beijerinckiaceae* were mainly found in the phyllosphere, reflecting the niche preference of these taxa. The family *Flavobacteriaceae* was exclusively found in the rhizosphere and soil, while *Moraxellaceae* and *Rhizobiaceae* were exclusive for phyllosphere compartments and the rhizosphere.

The most abundant fungal family was *Mycosphaerellaceae* (overall average abundance: 14.9%), and was present with average abundances 43.3%, 0.6% and 0.8% in the phyllosphere compartments, rhizosphere, and soil respectively. This was followed by *Mortierellaceae* (11.3%), and *unidentified Capnodiales* (10.5%) (Fig. 2d). The fungal families which were highly present in the rhizosphere and soil included *Chaetomiaceae* (average abundance: 5.8%), *Lasiosphaeriaceae* (3.4%), *Sordariomycetes-fam-Incertae-sedis* (2.8%), and *Psathyrellaceae* (2.6%). Analogous to the bacterial community, the soil and rhizosphere as compared to the phyllosphere compartments were associated with a high presence of fungal families classified as “others”, with relative abundances of 38.5%, 22.5%, and 7.5% respectively. Additionally, differences in microbiome composition between the rhizosphere and phyllopshere, as well as between strawberry cultivars were observed. These differences were mainly attributed to bacterial families *Flavobacteriaceae*, *Pseudomonadaceae*, *Moraxellaceae,* and *Micrococcaceae*. Meanwhile, some of the fungal taxa such as *Chaetomiaceae*, *Mycosphaerellaceae*, and unidentified *Capnodiales* varied in composition between cultivars.

### Strawberry cultivar influenced the structure and composition of phyllosphere microbiome

We observed the significant effect of host cultivar on microbial β-diversity among plant compartments (bacterial community: *P* = 0.023, R2 = 0.017; fungal community: *P* = 0.001, R2 = 0.023). Compartment-specific PCoA plots which were constructed using Bray Curtis distances, revealed cultivar separation in the various plant compartments (Additional File 1: Fig. S3 and S4), and statistical differences further confirmed by PERMANOVA (Additional File 1: Tables S3 and S4). The differences between strawberry cultivars were more pronounced in the phyllosphere compartments (bacteria-R^2^ = 16.8–36.5; fungi- R^2^ = 22.3 -91.1%) compared to rhizosphere (bacteria—R^2^ = 12.8; fungi—R^2^ = 14.2%) (Additional File 1: Tables S3 and S4). Therefore, comparative analysis of microbial community composition in the phyllosphere compartments for the two cultivars was performed.

**Fig. 3:**
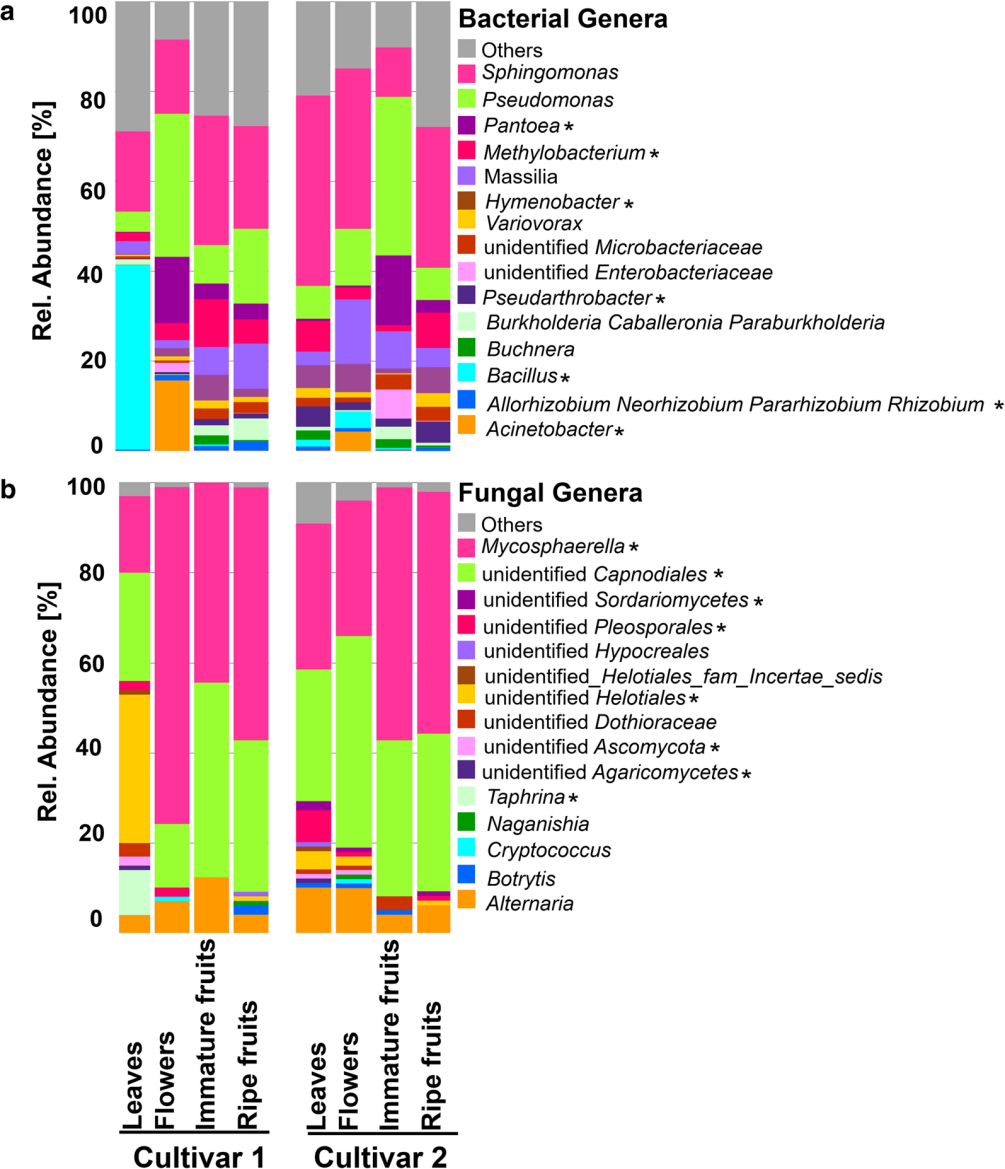
Microbial community comparison between strawberry cultivars in the different phyllosphere compartments. Bacterial (**a**) and fungal (**b**) communities are shown at genus level. The asterisks indicate genera which were significantly different as revealed by Linear discriminant analysis Effect Size (LEfSe). The stacked bar plots were based on top 15 genera, while all remaining taxa were included in “Others”

Microbial community composition within phyllosphere compartments, as well as between strawberry cultivars was visualized for the top 15 ASVs at genus level (Fig. 3). The most dominant bacterial taxa were identified as *Sphingomonas* (28.4%), *Pseudomonas* (9.6%), *Bacillus* (7.2%), *Massilia* (5.7%), *Pantoea* (5.2%), *Methylobacterium* (4.5%), and *Hymenobacter* (3.6%). These taxa showed variations among phyllosphere compartments as well as between cultivars. Predominant fungal genera included *Mycosphaerella* (average abundance: 42.7%), unidentified *Capnodiales* (31.3), *Alternaria* (6.6%), and unidentified *Helotiales* (5%). *Mycosphaerella* was found in all phyllosphere compartments, regardless of cultivar, and especially in flowers of cultivar 1 (Fig. 3b). A similar distribution was observed for *Botrytis*, it was present in ripe fruits of cultivar 1, as well as in leaves, immature fruits, and flowers of cultivar 2.

Linear discriminant analysis Effect Size (LEfSe) analysis performed on phyllosphere microbiomes revealed that 38 bacterial and 23 fungal genera were differentially abundant among phyllosphere microbiomes, as well as between cultivars. The bacterial and fungal genera with highest LDA scores (4 to 6.6) are shown in Additional File 1: Fig. S5a and b. *Bacillus*, *Pantoea*, *Burkholderia-Caballeronia-Paraburkholderia*, *Acinetobacter*, *Methylobacterium*, *Hymenobacterium*, and *Pseudarthrobacter* were among the bacterial genera which showed significant differential abundance, while fungal genera included *Mycosphaerella*, unidentified *Capnodiales*, unidentified *Helotiales*, *Taphrina*, unidentified *Pleosporales*, and unidentified *Dothioracea* (Fig. 3).

### Microbiome transfer between plant compartments during fruit development

**Fig. 4:**
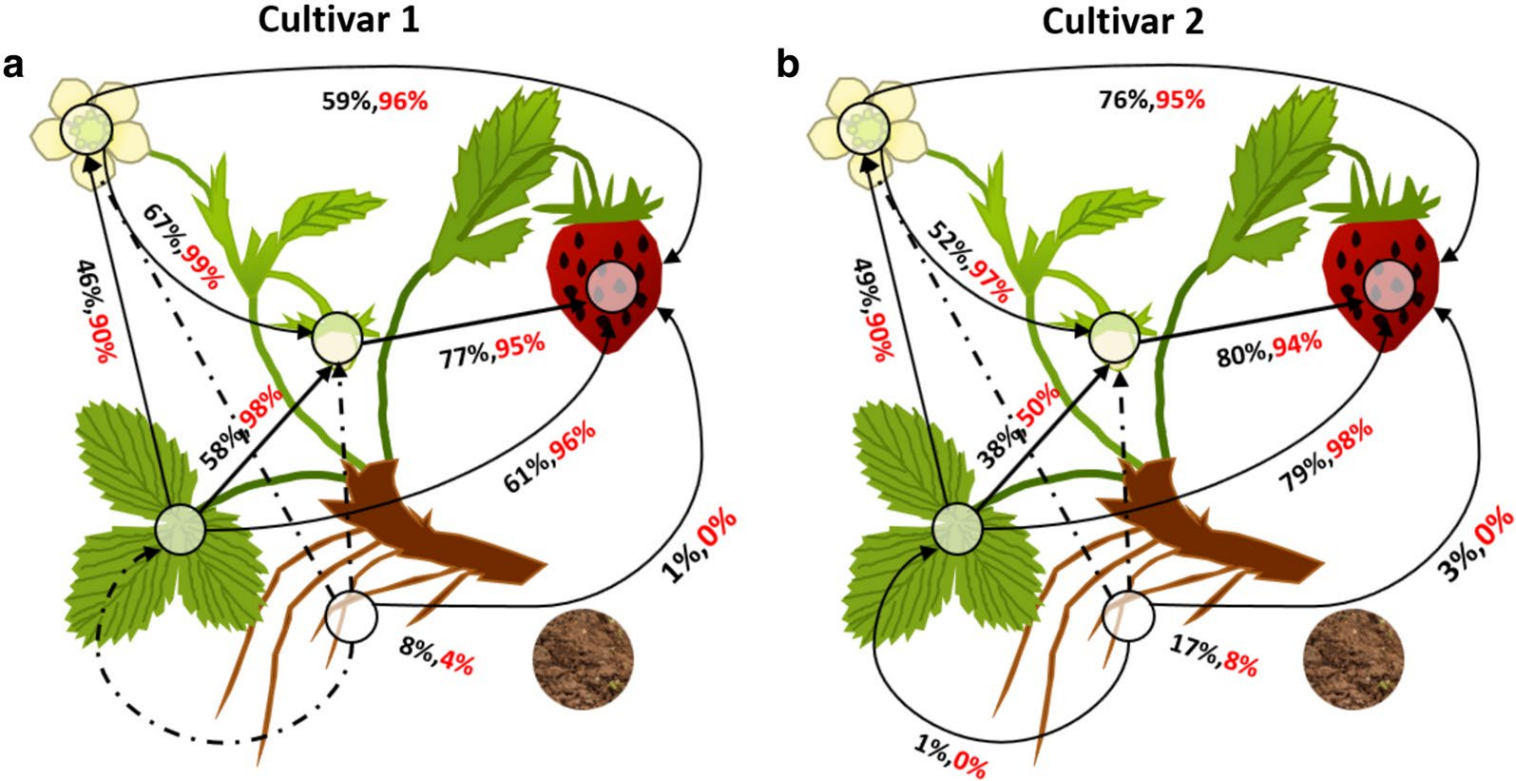
Tracking microbiome transfer between belowground and aboveground strawberry compartments. Cultivar 1 (**a**) and cultivar 2 (**b**) are shown separately. The compartments represented include bulk soil, rhizosphere, leaves, flowers, immature fruits and ripe fruits. The percentage of microbiota transferred is color coded to indicate bacteria (black) and fungi (red). Percentages given are based on SourceTracker2 analysis in R. The dotted lines represent source-sink linkages that showed no potential microbial transfer

The SourceTracker2 software was employed to study the microbiome that was potentially transferred between strawberry compartments. It revealed that within bacterial as well as fungal communities, low proportions of the microbiome were likely transferred from soil to the phyllosphere via the rhizosphere (Fig. 4). Moreover, direct microbiome transfer from soil to the phyllosphere was equally low (Additional File 1: Table S5). In contrast, a major microbiome transfer was identified from leaves, flowers, and immature fruits to ripe fruits, and from flowers and leaves into immature fruits. The proportion of fungal microbiota (50% to 99%) which were potentially transferred between phyllosphere compartments was substantially higher as compared to bacteria (38–80%). Generally, flowers, immature fruits and mature fruits shared the highest percentage of their microbiome; thus, indicating a major likely transfer of microbial signatures along the fruit development. In terms of cultivar, we found that cultivar 2 had a slightly higher enrichment of soil microbiota in the rhizosphere than cultivar 1, as well as a slightly higher transfer potential of rhizosphere-enriched bacteria to the phyllosphere compartments. Interestingly, either no or very little microbial transfer from belowground to the phyllosphere compartment was observed in both cultivars.

### Storage and disease state induced shift in fruit microbiome

**Fig. 5:**
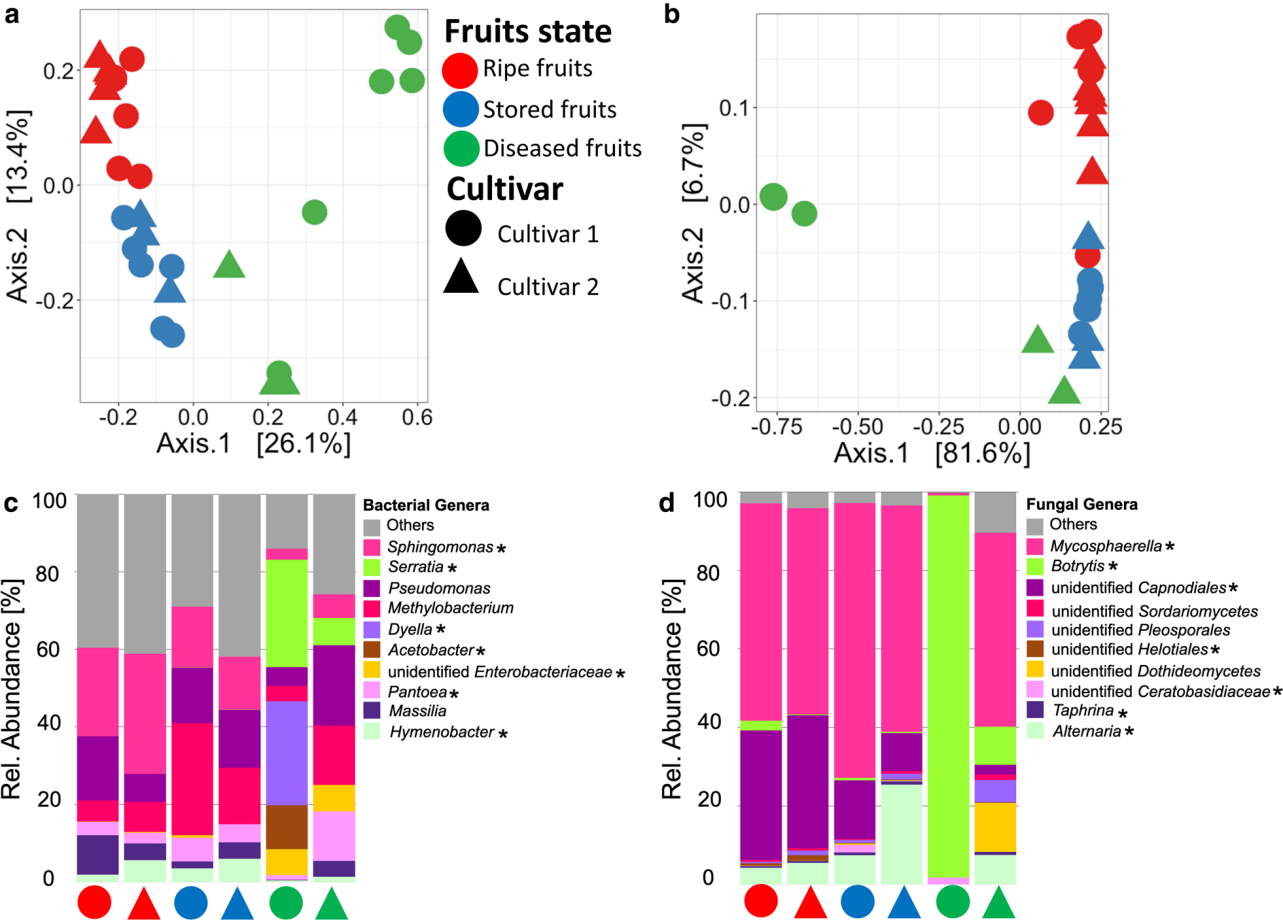
Microbial community structure and composition of fresh ripe fruits, stored fruits, and diseased fruits. Bacteria (**a** and **c**) and fungi (**b** and **d**) are respectively shown in PCoA and stacked bar plots representing the structure and composition of bacterial and fungal communities. The stacked bar plots were respectively based on the top 10 bacterial and fungal genera, while “Others” include the remaining taxa. The asterisks indicate genera which were significantly different among the three fruit conditions as determined by LEfSe. Colors represent fruit condition, while shapes show strawberry cultivar

To investigate the effect of storage and disease on the fruit microbiome, microbial shift analysis between fresh ripe fruits, stored fruits, and diseased fruits was performed. We observed that disease more than storage severely induced changes in the fruit microbiome. This was further supported by a significantly higher bacterial diversity in ripe fruits as compared to stored and diseased fruits (Fig. 1, Additional File 1: Tables S3, and S4); the fungal community, on the other hand, showed no significant differences. We also noticed an increase in microbial abundance and a simultaneous decrease in diversity in stored and diseased fruits; however, the highest shifts were observed for diseased fruits (Fig. 1, Additional File 1: Tables S2, S3, and S4). For example, the disease condition increased bacterial and fungal abundance by 60% and 80% respectively, while decreasing diversity by 29% and 48%. In addition, storage increased bacterial and fungal abundance by 54% and 40% respectively, while decreasing diversity by 1.5% and 2.8%. Interestingly, this shift in microbiome composition and separation was also observed among the strawberry cultivars as shown in Fig. 5. Statistical analysis based on PERMANOVA revealed a significant effect of fruit condition (*P* = 0.001) on microbial community composition (R^2^ = 33 and 66% for bacterial and fungal communities, respectively). Likewise, the significant effect of cultivar on the different fruit conditions, as well as their interactions was shown (Additional file 1: Table S6). PCoA visualization of Bray–Curtis distances further showed community separation among fruit conditions and cultivar (Fig. 5a, b). Moreover, changes in taxonomic composition were also evident. The abundance of *Proteobacteria* in ripe fruits, stored and diseased fruits showed substantial variations (69.5%, 78.6% and 94.7%, respectively) as did that of *Actinobacteria* (18.2%, 8.5% and 1.1%, respectively); while *Firmicutes* were highly abundant in stored fruits (3.3%). Conversely, for the fungal community, *Ascomycota* (97.9%) and *Basidiomycota* (2%) were dominant.

On genus level, *Sphingomonas* (average abundance: 15.5%), *Methylobacterium* (13.2%), and *Pseudomonas* (11.4%) were predominant; *Sphingomonas* was highly present in ripe fruits followed by stored fruits, while *Methylobacterium* (23.7%) was accumulated in stored fruits (Fig. 5c). Significant differentially abundant bacterial genera among fruit conditions and strawberry cultivars (LEfSE analysis) included: *Hymenobacter* (enriched in ripe and stored fruits); *Massilia* (ripe fruits); as well as *Dyella*, *Acetobacter*, and *Serratia* (were exclusively found associated with diseased fruits) as shown in Additional File 1: Fig. S5c. Fungal genera including *Mycosphaerella* (average abundance: 45.4%), *Botrytis* (21.8%), *unidentified Capnodiales* (15.7%), and *Alternaria* (6.4%), predominated the microbiomes of ripe fruits, stored fruits, and diseased fruits. Remarkably, *Botrytis* (84%) was highly present in diseased fruits, while *Mycosphaerella* (65%) and *Alternaria* (15%) were enriched in stored fruits (Fig. 5d). Significant differential abundance was found for: *Mycosphaerella, Botrytis,* unidentified *Capnodiales,* unidentified *Helotiales,* unidentified *Ceratobasidiaceae, Taphrina,* and *Alternaria* as shown in Additional file 1: Fig.S5d*.* Our attempt to visualize bacteria colonizing the strawberry fruit tissues revealed the presence of bacteria in the fruit pulp and the outer fruit surface (Additional File 1: Fig. S6); all bacteria were labeled with the universal FISH probes included in EUB338MIX. However, we were unable to visualize bacteria corresponding to *Firmicutes* and *Gammaproteobacteria* labelled with specific probes (Cy5-labeled LGC-mix and ALEXA488-labeled GAM42a, respectively).

## Discussion

In the present study, we observed a specific microbial composition in the phyllosphere and belowground compartments with a higher belowground diversity and abundance similar to other plant hosts [6, 16, 72]. Plant compartments were found to have a greater impact on the plant microbiome than cultivars, which is consistent with prior findings [73].

Microbiome assembly in the phyllosphere and rhizosphere of strawberry plants was significantly different, and especially the phyllosphere showed a clear developmental pattern. During fruit development, from flower through immature fruit to ripe fruit, we found that a substantial fraction of the microbiome is shared between those compartments indicating a major transfer of communities along that route. Fruit development was also associated with an increase of bacterial diversity and abundance. This could partly be attributed to the cumulative occupation of different microorganisms during fruit development. Moreover, microbiota transmission via insects and wind during pollination was previously suggested to contribute to plant and fruit microbiome assembly [74]. Occurrence of the genus *Buchnera* in strawberry, which is strongly associated with insects, is an indication for that observation. In addition, the growth of strawberry stolons in proximity to the ground, could have potentially contributed to the transfer of soil microorganisms to the fruit. Bacterial taxa including *Sphingomonas*, *Pseudomonas*, *Massilia, Pantoea*, *Acinetobacter*, *Methylobacterium*, and *Hymenobacter* were prevalent in the phyllosphere microbiomes including flowers and fruits. *Sphingomonas* and *Pseudomonas* were previously found to be abundant in apple flower [75] and fruit [76] microbiomes. Moreover, phyllosphere-adapted bacterial genera like *Methylobacterium* and *Sphingomonas* [2, 77] are known to commonly compose the core of phyllosphere microbiomes where they can have implications for plant growth promotion and pathogen suppression [11, 78–80], and even for strawberry flavor [12]. S*phingomonas* was previously shown to have a high niche versatility in the phyllosphere; it was detected in the core microbiomes of tomato trichomes as well as apple fruits [76, 81]. On the other hand, for the fungal community, a decrease in diversity was observed during fruit development. Genera including *Mycosphaerella* and *Alternaria*, as well as *unidentified Capnodiales* and *unidentified Helotiales* were predominant as previously observed [27, 82]. *Mycosphaerella* is the largest genus of ascomycetes and clades of this genus representing *Cercospora* and *Septoria* are known plant pathogens [83, 84]. *Alternaria* and *Mycosphaerella* are both leaf-infecting fungi, and *Alternaria* has been linked to common human allergens in addition to being a plant pathogen [85, 86]. Moreover, *Cryptococcus*, a yeast previously isolated from fruit washings and used in biological management of fruit postharvest diseases [87], was found to be associated with flowers and potentially transferred to the fruit microbiome. However, this needs further attention as some members of the genus *Cryptococcus* have been associated with potential health hazards to human and animal health [88, 89].

Further decipherment of the fruit microbiome revealed distinct shifts due to cold storage and disease incidence. Generally, for either community, a higher microbial diversity was observed in fresh ripe fruits compared to diseased fruits. Simultaneously, we observed an increase in bacterial and fungal abundances (up to 80%) in stored and diseased fruits in comparison to ripe fruits. The decrease in diversity due to disease has previously been associated to the so- called “dysbiosis” state [33, 90]. Fruits with higher diversity are thought to be healthier as well as better storable, while dysbiosis is often linked to predominance of opportunistic taxa [33]. In spite of the comparably similar microbiome compositions between ripe and stored fruits, the proportion of genera such as *Shingomonas* decreased in stored fruits. We presume that the reduction in abundance of potentially core members like *Sphingomonas* is connected to disruptions, favoring opportunists or even pathogenic taxa as previously revealed [91]. Alterations in microbial networks as well as reduced diversity are commonly associated with disease outbreaks [1]. In the present study, the diseased fruit microbiome was associated with a high occurrence of distinct taxa including *Serratia*, *Pantoea*, *Dyella*, *Pseudomonas*, and unidentified *Enterobacteriaceae*. Members of the family *Enterobacteriaceae* found in edible plants may contain taxa associated with food health and safety [92, 93]. Interestingly, genera like *Serratia* and *Pseudomonas,* which are potentially beneficial for plant health and growth [35, 94], were also identified in diseased fruits. The presence of these beneficial taxa in diseased fruits could be due to accumulation of antagonists against *Botrytis* which was found to be predominant in diseased fruits. The fungal community in ripe and stored fruits was characterized by differences in the composition of *Mycosphaerella* and *Alternaria* in accordance to earlier observations [27]; however, higher abundances were observed in stored fruits.

The similarity between the aboveground and belowground compartiments was low, and only a low proportion of the microbiome was shown to be potentially transferred from soil via the rhizosphere to the phyllosphere. Moreover, direct microbiome transfer from soil to the phyllosphere compartments was equally low, but much lower in comparison to the rhizosphere-phyllosphere route. The identified unexpected high difference in microbial diversity between soil and the rhizosphere in this study could be attributed to differences between environmental soil and soil associated with seedlings from the plant nursery. Overall, our study highlights the compartment-specificity of microbial communities and the simultaneous strong connectivity between them. Moreover, it shows that the fruit microbiome assembly mainly depends on precursor stages of fruit development with lower implications of other compartments. The plant microbiome, shaped by biotic and abiotic factors, accompanies the plant throughout its whole life cycle [95]. Particularly in the phyllopshere, the microbiome has been found to be influenced by plant genetics, solar radiation, temperature, and humidity which can partially explain the observed spatio-temporal variations [8, 91]. Plant communities are critical to plant health; however, microbial communities on freshly consumed produce, such as strawberries, are also of interest in terms of their relationship to human health [93]. Altogether, calculation of abundance revealed approximately 2 billion of microorganisms in fresh ripe fruits of average weight. We note that this was based on qPCR amplifications without specific blockers for plant organelle DNA. In addition, FISH-CLSM visualization of bacteria revealed some bacteria resident in the fruit pulp; however, owing to technical challenges, our effort to visualize bacteria belonging to classes *Firmicutes* and *Gammaproteobacteria* were not successful. We demonstrated that freshly harvested strawberries have higher microbial diversity compared to stored and diseased strawberries, although the fungal community diversity was not significantly different. This indicates a potential health benefit of consuming freshly harvested produce in terms of enriching the gut microbiome and stimulating the immune system. Microbiome shifts induced by cold storage might reduce such effects.

## Conclusions

A deep understanding of plant microbiome assembly could further provide the basis to enhance fruit microbial communities already while they are formed, as well as facilitate the development of potential postharvest biocontrol agents. Healthier and more diverse fruit microbiomes could potentially decrease disease susceptibility as well as increase storability of fruits. Investigation of the strawberry plant microbiome revealed that significant microbial fractions are transferred between different compartments during developmental stages; particularly within the phyllosphere compartments. During fruit development, the microbiome was gradually enriched from precursor stages to ripe fruits. While postharvest storage only induced minor shifts in the fruit microbiome, disease occurrence resulted in substantial shifts mainly characterized by a reduction in microbial diversity and an increase of potentially pathogenic taxa. Future applications such as phyllosphere application of bioinoculants could make use of these findings to introduce microbiome shifts already during fruit development to obtain better storable and healthier fruits.

## Supplementary Information

**Additional file 1**. Supplementary information including additional statistical support for alpha and beta diversity measures as well as confocal laser scanning microscopy micrographs.

## Data Availability

The dataset supporting the conclusions of this article is available in the European Nucleotide Archive (ENA) (http://www.ebi.ac.uk/ena) under the project number PRJEB47398.

## Abbreviations

ASV: Amplicon Sequence Variants
DNA: Deoxyribonucleic acid
rRNA: Ribosomal Ribonucleic Acid
FISH-CLSM: Fluorescence in situ hybridization and confocal laser scanning microscopy

## References

[1] Berg G, Rybakova D, Fischer D, Cernava T, Vergès MCC, Charles T, et al. Microbiome definition re-visited: old concepts and new challenges. Microbiome. 2020;8. 10.1186/s40168-020-00875-0 PMC7329523 32605663

[2] Bulgarelli D, Schlaeppi K, Spaepen S, Ver E, Van Themaat L, Schulze-Lefert P. Structure and functions of the bacterial microbiota of plants. 2013. doi:10.1146/annurev-arplant-050312-120106. 10.1146/annurev-arplant-050312-120106 23373698

[3] Abdelfattah A, Wisniewski M, Schena L, Tack AJM (2021). Experimental evidence of microbial inheritance in plants and transmission routes from seed to phyllosphere and root. Environ Microbiol.

[4] Berg G, Raaijmakers JM (2018). Saving seed microbiomes. ISME J.

[5] McCully ME. Niches for bacterial endophytes in crop plants: a plant biologist’s view. In: Australian Journal of Plant Physiology. CSIRO; 2001. p. 983–90. doi:10.1071/pp01101.

[6] Vorholt JA (2012). Microbial life in the phyllosphere. Nat Rev Microbiol.

[7] Berg G, Krechel A, Ditz M, Sikora RA, Ulrich A, Hallmann J (2005). Endophytic and ectophytic potato-associated bacterial communities differ in structure and antagonistic function against plant pathogenic fungi. FEMS Microbiol Ecol.

[8] F B, I C. Pivotal roles of phyllosphere microorganisms at the interface between plant functioning and atmospheric trace gas dynamics. Front Microbiol. 2015. doi:10.3389/FMICB.2015.00486. 10.3389/fmicb.2015.00486 PMC4440916 26052316

[9] Lebeis SL (2015). Greater than the sum of their parts: characterizing plant microbiomes at the community-level. Curr Opin Plant Biol.

[10] Berendsen RL, Pieterse CMJ, Bakker PAHM (2012). The rhizosphere microbiome and plant health. Trends Plant Sci.

[11] Matsumoto H, Fan X, Wang Y, Kusstatscher P, Duan J, Wu S (2021). Bacterial seed endophyte shapes disease resistance in rice. Nat Plants.

[12] Verginer M, Leitner E, Berg G (2010). Production of odor-active metabolites by grape-associated microorganisms. J Agric Food Chem.

[13] Schmidt R, Köberl M, Mostafa A, Ramadan EM, Monschein M, Jensen KB, et al. Effects of bacterial inoculants on the indigenous microbiome and secondary metabolites of chamomile plants. Front Microbiol. 2014;5 FEB:64. doi:10.3389/fmicb.2014.00064. 10.3389/fmicb.2014.00064 PMC3928675 24600444

[14] Turner TR, James EK, Poole PS (2013). The plant microbiome. Genome Biol.

[15] Raaijmakers JM, Paulitz TC, Steinberg C, Alabouvette C, Moënne-Loccoz Y (2009). The rhizosphere: a playground and battlefield for soilborne pathogens and beneficial microorganisms. Plant Soil.

[16] Philippot L, Raaijmakers JM, Lemanceau P, Van Der Putten WH (2013). Going back to the roots: the microbial ecology of the rhizosphere. Nat Rev Microbiol.

[17] Vandenkoornhuyse P, Quaiser A, Duhamel M, Le Van A, Dufresne A (2015). The importance of the microbiome of the plant holobiont. New Phytol.

[18] Todeschini V, AitLahmidi N, Mazzucco E, Marsano F, Gosetti F, Robotti E (2018). Impact of beneficial microorganisms on strawberry growth, fruit production, nutritional quality, and volatilome. Front Plant Sci.

[19] Schieberle P, Hofmann T (1997). Evaluation of the character impact odorants in fresh strawberry juice by quantitative measurements and sensory studies on model mixtures. J Agric Food Chem.

[20] Ulrich D, Hoberg E, Rapp A, Kecke S (1997). Analysis of strawberry flavour - discrimination of aroma types by quantification of volatile compounds. Eur Food Res Technol.

[21] Schwieterman ML, Colquhoun TA, Jaworski EA, Bartoshuk LM, Gilbert JL, Tieman DM (2014). Strawberry flavor: diverse chemical compositions, a seasonal influence, and effects on sensory perception. PLoS ONE.

[22] Lila MA (2004). Anthocyanins and human health: an in vitro investigative approach. J Biomed Biotechnol.

[23] Khoo HE, Azlan A, Tang ST, Lim SM. Anthocyanidins and anthocyanins: Colored pigments as food, pharmaceutical ingredients, and the potential health benefits. Food Nutr Res. 2017;61. doi:10.1080/16546628.2017.1361779. 10.1080/16546628.2017.1361779 PMC5613902 28970777

[24] Horbowicz M, Grzesiuk A, DĘBski H, Kosson R. Anthocyanins of Fruits and Vegetables - Their Occurrence, Analysis and Role in Human. Veg Crop Res Bull. 2008;68:5–22.

[25] Li D, Wang P, Luo Y, Zhao M, Chen F (2017). Health benefits of anthocyanins and molecular mechanisms: update from recent decade. Crit Rev Food Sci Nutr.

[26] FAOSTAT. Food and Agriculture Organization of the United Nations. FAOSTAT database. 2019. http://www.fao.org/faostat/en/#data/QC. Accessed 8 Jun 2021.

[27] Abdelfattah A, Wisniewski M, Li Destri Nicosia MG, Cacciola SO, Schena L. Metagenomic Analysis of fungal diversity on strawberry plants and the effect of management practices on the fungal community structure of aerial organs. PLoS One. 2016;11:e0160470. doi:10.1371/journal.pone.0160470. 10.1371/journal.pone.0160470 PMC4973904 27490110

[28] Xu X, Passey T, Wei F, Saville R, Harrison RJ. Amplicon-based metagenomics identified candidate organisms in soils that caused yield decline in strawberry. Hortic Res. 2015;2 March. 10.1038/hortres.2015.22 PMC4595980 26504572

[29] Heydari A, Pessarakli M (2010). A review on biological control of fungal plant pathogens using microbial antagonists. J Biol Sci.

[30] Berg G, Kurze S, Buchner A, Wellington EM, Smalla K (2000). Successful strategy for the selection of new strawberry-associated rhizobacteria antagonistic to *Verticillium* wilt. Can J Microbiol.

[31] Kurze S, Bahl H, Dahl R, Berg G (2001). Biological control of fungal strawberry diseases by Serratia plymuthica HRO-C48. Plant Dis.

[32] Zabetakis I (1997). Enhancement of flavour biosynthesis from strawberry (Fragaria × ananassa) callus cultures by *Methylobacterium* species. Plant Cell Tissue Organ Cult.

[33] Kusstatscher P, Cernava T, Abdelfattah A, Gokul J, Korsten L, Berg G. Microbiome approaches provide the key to biologically control postharvest pathogens and storability of fruits and vegetables. FEMS Microbiol Ecol. 2020;96. 10.1093/femsec/fiaa119 32542314

[34] Smalla K, Wieland G, Buchner A, Zock A, Parzy J, Kaiser S (2001). Bulk and rhizosphere soil bacterial communities studied by denaturing gradient gel electrophoresis: plant-dependent enrichment and seasonal shifts revealed. Appl Environ Microbiol.

[35] Berg G, Roskot N, Steidle A, Eberl L, Zock A, Smalla K (2002). Plant-dependent genotypic and phenotypic diversity of antagonistic rhizobacteria isolated from different *Verticillium* host plants. Appl Environ Microbiol.

[36] Deng S, Wipf HML, Pierroz G, Raab TK, Khanna R, Coleman-Derr D (2019). A plant growth-promoting microbial soil amendment dynamically alters the strawberry root bacterial microbiome. Sci Rep.

[37] De Tender C, Haegeman A, Vandecasteele B, Clement L, Cremelie P, Dawyndt P, et al. Dynamics in the strawberry rhizosphere microbiome in response to biochar and *Botrytis cinerea* Leaf Infection. Front Microbiol. 2016; 2062. doi:10.3389/fmicb.2016.02062. 10.3389/fmicb.2016.02062 PMC5177642 28066380

[38] SAD DK-, Sadjarstvo R za, Vinarstvo V in, 2010 U. Mara des Bois-a storybook strawberry. cabdirect.org. 2010. https://www.cabdirect.org/cabdirect/abstract/20103334055. Accessed 9 Jun 2021.

[39] Allais I, Létang G. Influence of mist-chilling on post-harvest quality of fresh strawberries Cv. Mara des Bois and Gariguette. Int J Refrig. 2009;32:1495–504.

[40] Kaiser R, Mageney V, Schwefel K, Vollmers D, Krüger A, Horn R. Genotyping of red and white fruited strawberry (*Fragaria* L.) accessions and hybrids based on microsatellite markers and on the genetic diversity in the allergen genes fra a 1 and fra a 3. Genet Resour Crop Evol. 2016;63:1203–17. doi:10.1007/s10722-015-0311-x.

[41] Franz-Oberdorf K, Eberlein B, Edelmann K, Bleicher P, Kurze E, Helm D (2017). White-fruited strawberry genotypes are not per se hypoallergenic. Food Res Int.

[42] Köberl M, Müller H, Ramadan EM, Berg G. Desert farming benefits from microbial potential in arid soils and promotes diversity and plant health. PLoS One. 2011;6. 10.1371/journal.pone.0024452 PMC3166316 21912695

[43] White TJ, Bruns T, Lee S, Taylor J. Amplification and Direct Sequencing of Fungal Ribosomal Rna Genes for Phylogenetics. PCR Protoc. 1990; January:315–22.

[44] Caporaso JG, Lauber CL, Walters WA, Berg-Lyons D, Lozupone CA, Turnbaugh PJ (2011). Global patterns of 16S rRNA diversity at a depth of millions of sequences per sample. Proc Natl Acad Sci U S A.

[45] Caporaso JG, Lauber CL, Walters WA, Berg-Lyons D, Huntley J, Fierer N (2012). Ultra-high-throughput microbial community analysis on the Illumina HiSeq and MiSeq platforms. ISME J.

[46] Peiffer JA, Spor A, Koren O, Jin Z, Tringe SG, Dangl JL (2013). Diversity and heritability of the maize rhizosphere microbiome under field conditions. Proc Natl Acad Sci U S A.

[47] Lundberg DS, Yourstone S, Mieczkowski P, Jones CD, Dangl JL (2013). Practical innovations for high-throughput amplicon sequencing. Nat Methods.

[48] Fitzpatrick CR, Lu-Irving P, Copeland J, Guttman DS, Wang PW, Baltrus DA (2018). Chloroplast sequence variation and the efficacy of peptide nucleic acids for blocking host amplification in plant microbiome studies. Microbiome.

[49] GARDES M, BRUNS TD. ITS primers with enhanced specificity for basidiomycetes - application to the identification of mycorrhizae and rusts. Mol Ecol. 1993;2:113–8. doi:10.1111/j.1365-294X.1993.tb00005.x. 10.1111/j.1365-294x.1993.tb00005.x 8180733

[50] Martin M. Cutadapt removes adapter sequences from high-throughput sequencing reads. EMBnet.journal. 1994;17:10–2. https://journal.embnet.org/index.php/embnetjournal/article/view/200/479.

[51] Bolyen E, Rideout JR, Dillon MR, Bokulich NA, Abnet CC, Al-Ghalith GA (2019). Reproducible, interactive, scalable and extensible microbiome data science using QIIME 2. Nat Biotechnol.

[52] Callahan BJ, McMurdie PJ, Rosen MJ, Han AW, Johnson AJA, Holmes SP (2016). DADA2: high-resolution sample inference from Illumina amplicon data. Nat Methods.

[53] Quast C, Pruesse E, Yilmaz P, Gerken J, Schweer T, Yarza P (2013). The SILVA ribosomal RNA gene database project: improved data processing and web-based tools. Nucleic Acids Res.

[54] Yilmaz P, Parfrey LW, Yarza P, Gerken J, Pruesse E, Quast C (2014). The SILVA and “all-species Living Tree Project (LTP)” taxonomic frameworks. Nucleic Acids Res.

[55] Abarenkov K, Nilsson RH, Larsson K-H, Alexander IJ, Eberhardt U, Erland S (2010). The UNITE database for molecular identification of fungi: recent updates and future perspectives. New Phytol.

[56] R Core Team. R: A language and environment for statistical computing. R Foundation for Statistical Computing, Vienna, Austria. URL https://www.R-project.org/. 2020. https://stat.ethz.ch/R-manual/R-devel/library/utils/html/citation.html. Accessed 8 May 2021.

[57] Dhariwal A, Chong J, Habib S, King IL, Agellon LB, Xia J (2017). MicrobiomeAnalyst: a web-based tool for comprehensive statistical, visual and meta-analysis of microbiome data. Nucleic Acids Res.

[58] McMurdie PJ, Holmes S. Phyloseq: an R Package for reproducible interactive analysis and graphics of microbiome census data. PLoS One. 2013;8. 10.1371/journal.pone.0061217 PMC3632530 23630581

[59] Oksanen J, Blanchet FG, Friendly M, Kindt R, Legendre P, Mcglinn D, et al. Package “vegan” Title Community Ecology Package Version 2.5–7. 2020.

[60] Kandlikar GS, Gold ZJ, Cowen MC, Meyer RS, Freise AC, Kraft NJB, et al. ranacapa: An R package and Shiny web app to explore environmental DNA data with exploratory statistics and interactive visualizations. F1000Research 2018 71734. 2018;7:1734. doi:10.12688/f1000research.16680.1. 10.12688/f1000research.16680.1 PMC6305237 30613396

[61] Segata N, Izard J, Waldron L, Gevers D, Miropolsky L, Garrett WS (2011). Metagenomic biomarker discovery and explanation. Genome Biol.

[62] Knights D, Kuczynski J, Charlson ES, Zaneveld J, Mozer MC, Collman RG (2011). Bayesian community-wide culture-independent microbial source tracking. Nat Methods.

[63] Abdelfattah A, Sanzani SM, Wisniewski M, Berg G, Cacciola SO, Schena L (2019). Revealing cues for fungal interplay in the plant-air interface in vineyards. Front Plant Sci.

[64] Cardinale M, Vieira De Castro J, Müller H, Berg G, Grube M. In situ analysis of the bacterial community associated with the reindeer lichen *Cladonia* arbuscula reveals predominance of Alphaproteobacteria. FEMS Microbiol Ecol. 2008;66:63–71. 10.1111/j.1574-6941.2008.00546.x 18631179

[65] Daims H, Brühl A, Amann R, Schleifer KH, Wagner M (1999). The domain-specific probe EUB338 is insufficient for the detection of all bacteria: development and evaluation of a more comprehensive probe set. Syst Appl Microbiol.

[66] Mahmoud KK, McNeely D, Elwood C, Koval SF (2007). Design and performance of a 16S rRNA-targeted oligonucleotide probe for detection of members of the genus *Bdellovibrio* by fluorescence in situ hybridization. Appl Environ Microbiol.

[67] Meier H, Amann R, Ludwig W, Schleifer KH (1999). Specific oligonucleotide probes for in situ detection of a major group of gram-positive bacteria with low DNA G+C content. Syst Appl Microbiol.

[68] Manz W, Amann R, Ludwig W, Wagner M, Schleifer KH (1992). Phylogenetic Oligodeoxynucleotide probes for the major subclasses of *Proteobacteria*: problems and solutions. Syst Appl Microbiol.

[69] Gonçalves AB, Santos IM, Paterson RRM, Lima N (2006). FISH and Calcofluor staining techniques to detect in situ filamentous fungal biofilms in water. Rev Iberoam Micol.

[70] Větrovský T, Baldrian P (2013). The variability of the 16S rRNA gene in bacterial genomes and its consequences for bacterial community analyses. PLoS ONE.

[71] Lofgren LA, Uehling JK, Branco S, Bruns TD, Martin F, Kennedy PG (2019). Genome-based estimates of fungal rDNA copy number variation across phylogenetic scales and ecological lifestyles. Mol Ecol.

[72] Hardoim PR, van Overbeek LS, Berg G, Pirttilä AM, Compant S, Campisano A (2015). The hidden world within plants: ecological and evolutionary considerations for defining functioning of microbial endophytes. Microbiol Mol Biol Rev.

[73] Wei N, Ashman TL. The effects of host species and sexual dimorphism differ among root, leaf and flower microbiomes of wild strawberries in situ. Sci Rep. 2018;8. 10.1038/s41598-018-23518-9 PMC5979953 29581521

[74] Griggs RG, Steenwerth KL, Mills DA, Cantu D, Bokulich NA (2021). Sources and assembly of microbial communities in vineyards as a functional component of winegrowing. Front Microbiol.

[75] Shade A, McManus PS, Handelsman J. Unexpected diversity during community succession in the apple flower microbiome. MBio. 2013;4. doi:10.1128/mBio.00602-12. 10.1128/mBio.00602-12 PMC3585449 23443006

[76] Wassermann B, Müller H, Berg G (2019). An apple a day: which bacteria do we eat with organic and conventional apples?. Front Microbiol.

[77] Delmotte N, Knief C, Chaffron S, Innerebner G, Roschitzki B, Schlapbach R (2009). Community proteogenomics reveals insights into the physiology of phyllosphere bacteria. Proc Natl Acad Sci U S A.

[78] Janakiev T, Dimkić I, Unković N, Ljaljević Grbić M, Opsenica D, Gašić U (2019). Phyllosphere fungal communities of plum and antifungal activity of indigenous phenazine-producing pseudomonas synxantha against monilinia laxa. Front Microbiol.

[79] Taffner J, Laggner O, Wolfgang A, Coyne D, Berg G (2020). Exploring the microbiota of east african indigenous leafy greens for plant growth, health, and resilience. Front Microbiol.

[80] Cui Z, Huntley RB, Schultes NP, Steven B, Zeng Q. Inoculation of stigma-colonizing microbes to apple stigmas alters microbiome structure and reduces the occurrence of fire blight disease. 2021. doi:10.1094/PBIOMES-04-20-0035-R.

[81] Kusstatscher P, Wicaksono WA, Bergna A, Cernava T, Bergau N, Tissier A (2020). Trichomes form genotype-specific microbial hotspots in the phyllosphere of tomato. Environ Microbiomes.

[82] Liu D, Howell K. Community succession of the grapevine fungal microbiome in the annual growth cycle. Environ Microbiol. 2020. 10.1111/1462-2920.15172 32686214

[83] Crous PW, Braun U, Groenewald JZ (2007). *Mycosphaerella* is polyphyletic. Stud Mycol.

[84] Quaedvlieg W, Kema GHJ, Groenewald JZ, Verkley GJM, Seifbarghi S, Razavi M, et al. *Zymoseptoria* gen. nov.: a new genus to accommodate Septoria-like species occurring on graminicolous hosts. Persoonia. 2011;26:57–69. doi:10.3767/003158511X571841. 10.3767/003158511X571841 PMC3160802 22025804

[85] Dang HX, Pryor B, Peever T, Lawrence CB (2015). The *Alternaria* genomes database: a comprehensive resource for a fungal genus comprised of saprophytes, plant pathogens, and allergenic species. BMC Genomics.

[86] Hernandez-ramirez G, Barber D, Tome-amat J, Garrido-arandia M, Diaz-perales A (2021). *Alternaria* as an inducer of allergic sensitization. J Fungi.

[87] Liu J, Sui Y, Wisniewski M, Droby S, Liu Y (2013). Review: utilization of antagonistic yeasts to manage postharvest fungal diseases of fruit. Int J Food Microbiol.

[88] May RC, Stone NRH, Wiesner DL, Bicanic T, Nielsen K (2016). *Cryptococcus*: from environmental saprophyte to global pathogen. Nat Rev Microbiol.

[89] Byrnes EJ, Bartlett KH, Perfect JR, Heitman J (2011). *Cryptococcus gattii*: an emerging fungal pathogen infecting humans and animals. Microbes Infect.

[90] Hooks KB, O’malley MA. Dysbiosis and its discontents. 2017. doi:10.1128/mBio.01492-17. 10.1128/mBio.01492-17 PMC5635691 29018121

[91] Liu H, Brettell LE, Singh B (2020). Linking the phyllosphere microbiome to plant health. Trends Plant Sci.

[92] Cernava T, Erlacher A, Soh J, Sensen CW, Grube M, Berg G. *Enterobacteriaceae* dominate the core microbiome and contribute to the resistome of arugula (*Eruca sativa* Mill.). Microbiome. 2019;7. doi:10.1186/s40168-019-0624-7. 10.1186/s40168-019-0624-7 PMC6352427 30696492

[93] Zhang H, Zhang Q, Chen S, Zhang Z, Song J, Long Z, et al. *Enterobacteriaceae* predominate in the endophytic microbiome and contribute to the resistome of strawberry. Sci Total Environ. 2020;727. 10.1016/j.scitotenv.2020.138708 32334231

[94] Lazcano C, Boyd E, Holmes G, Hewavitharana S, Pasulka A, Ivors K (2021). The rhizosphere microbiome plays a role in the resistance to soil-borne pathogens and nutrient uptake of strawberry cultivars under field conditions. Sci Rep.

[95] Edwards JA, Santos-Medellín CM, Liechty ZS, Nguyen B, Lurie E, Eason S (2018). Compositional shifts in root-associated bacterial and archaeal microbiota track the plant life cycle in field-grown rice. PLOS Biol.

