# Supplementary material for: Insights into the microbiome assembly during different growth stages and storage of strawberry plants
Source: Environ Microbiome. 2022 Apr 28;17:21. doi: 10.1186/s40793-022-00415-3 (PMC9052558; doi:10.1186/s40793-022-00415-3)
Supplement: Supplementary file 1 — Additional file 1. Supplementary information including additional statistical support for alpha and beta diversity measures as well as confocal laser scanning microscopy micrographs. [file 40793_2022_415_MOESM1_ESM.docx]

# Insights into the microbiome assembly during different growth stages and storage of strawberry plants

**Expedito Olimi, Peter Kusstatscher*, Wisnu Adi Wicaksono, Ahmed Abdelfattah, Tomislav Cernava & Gabriele Berg**

Institute of Environmental Biotechnology, Graz University of Technology, 8010 Graz, Austria

**
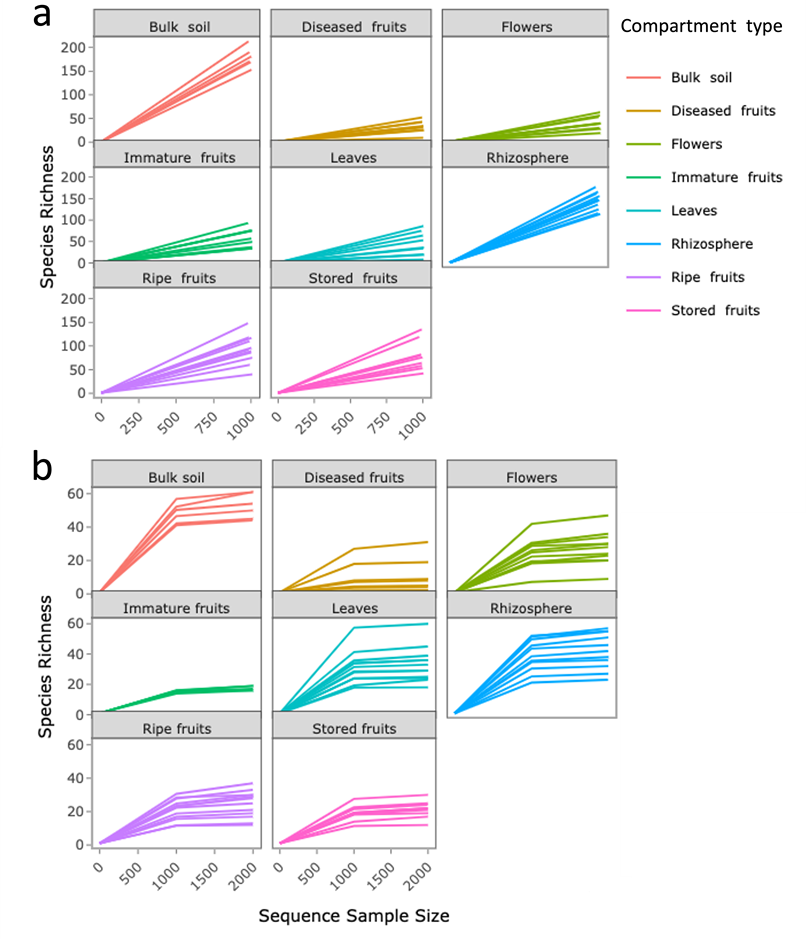
**

**Figure S1. Compartment-specific alpha rarefaction curves for Bacterial (a) and fungal communities (b).** The sampling depths for were 1000 and 2500 reads per sample for bacterial and fungal communities respectively. Colors represent different compartments.

**
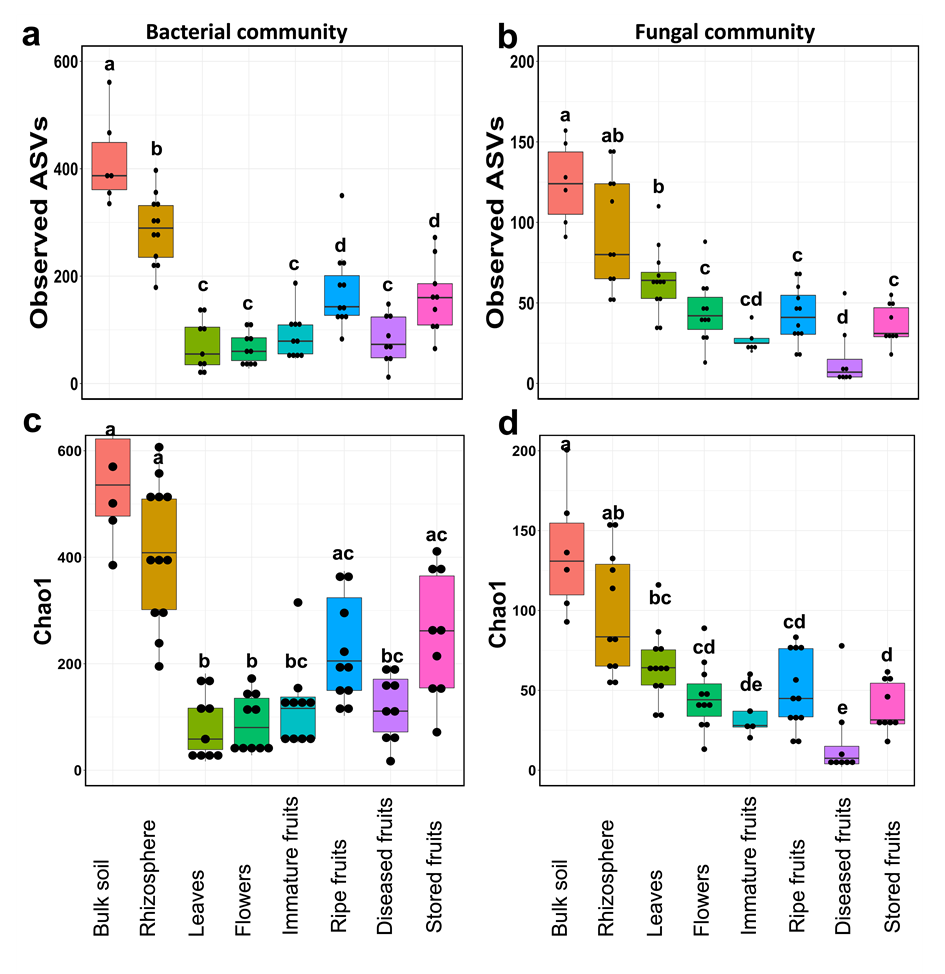
**

**Figure S2. Bacterial and fungal community richness as estimated by observed ASVs (a and b) and Chao1 (c and d).** Boxplots indicate bacterial (a, c) and fungal (b, d) richness in soil, rhizosphere and strawberry phyllosphere compartments. Significant differences (P<0.05) were respectively tested using pairwise Wilcoxon Rank Sum Test, followed by FDR correction. Statistical differences are indicated by different letters above the boxplots.


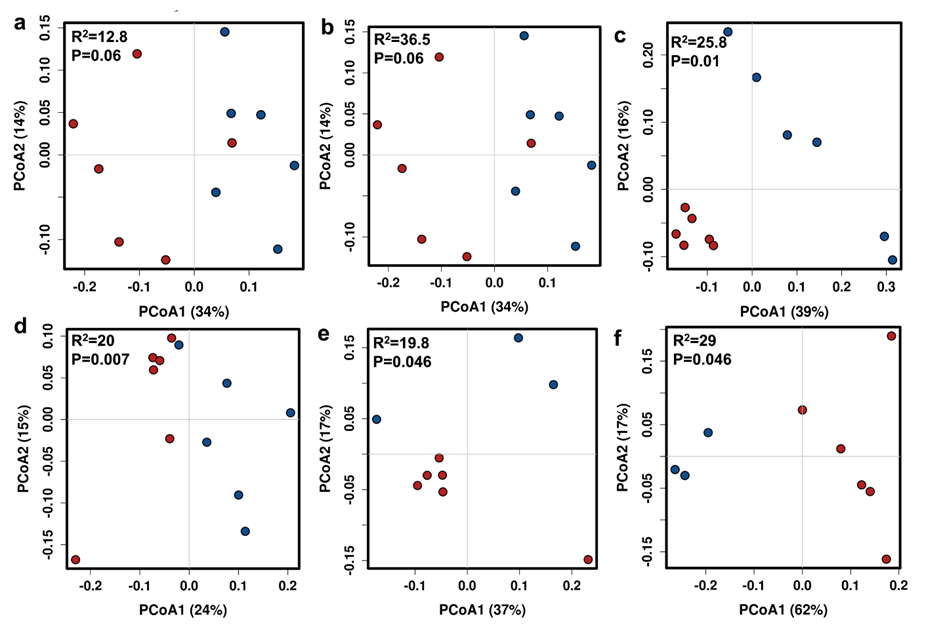


**Figure S3. PCoAs based on Bray-Curtis distances showing bacterial community separation in individual compartments.** The Bacterial community clustering in different compartments is represented as: rhizosphere (a), leaves (b), flowers (c), ripe fruits (d), stored fruits (e), and diseased fruits (f). No significant difference in microbial beta diversity was observed in immature fruits (PERMANOVA: R^2^=16.8 and P=0.05). Color codes on the PCoAs indicate host cultivars: cultivar 1 (red) and cultivar2 (blue).


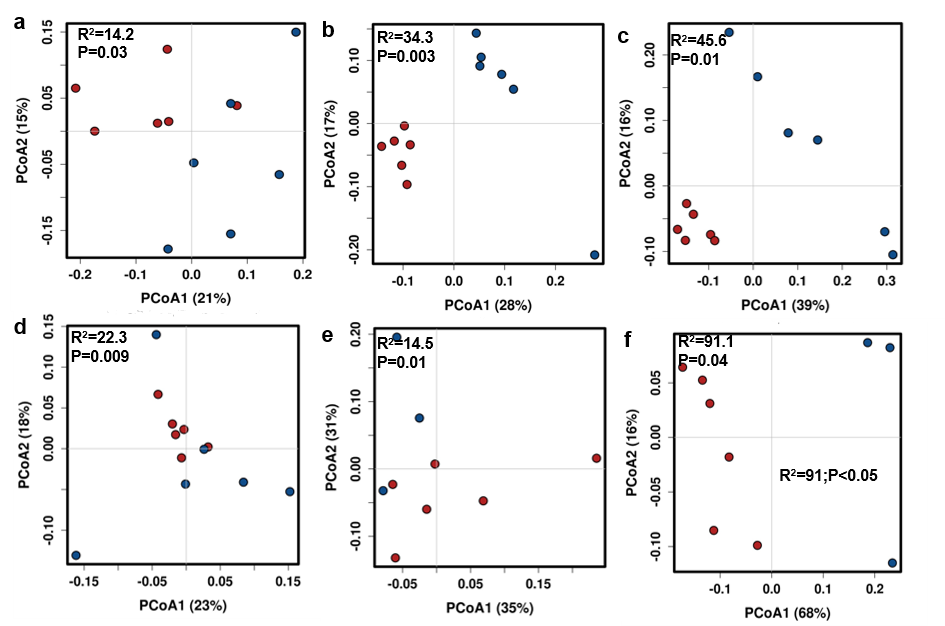


**Figure S4. PCoAs based on Bray-Curtis distances showing fungal community separation in individual compartments.** The fungal community clustering in different compartments is represented as: rhizosphere (a), leaves (b), flowers (c), ripe fruits (d), stored fruits (e), and diseased fruits (f). No significant difference in microbial beta diversity was observed in immature fruits (PERMANOVA: R^2^=32.8 and P=0.4). Color codes on the PCoAs indicate host cultivars: cultivar 1 (red) and cultivar2(blue).


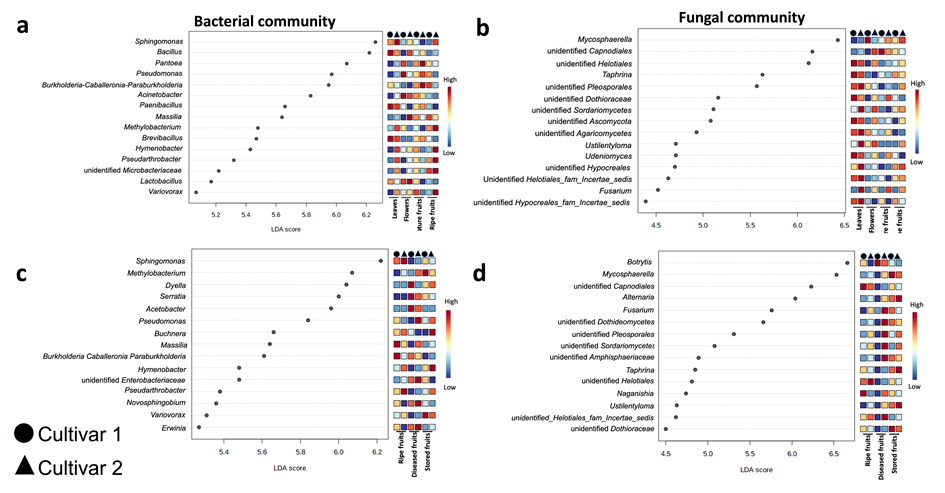


**Figure S5. Linear discriminant analysis Effect Size (LEfSe) plots showing genera explaining differences between strawberry cultivars in the different compartments and fruit conditions.** Plots (a and b) represent bacterial and fungal community differences between cultivars in the phyllosphere compartments, while (c and d) show cultivar differences between the fruit conditions. Feature table (ASV table) of subset datasets of fruit condition (c and d) and phyllosphere compartments (a and b), taxonomy, and mapping file were used as input. The shapes represent cultivars.

**
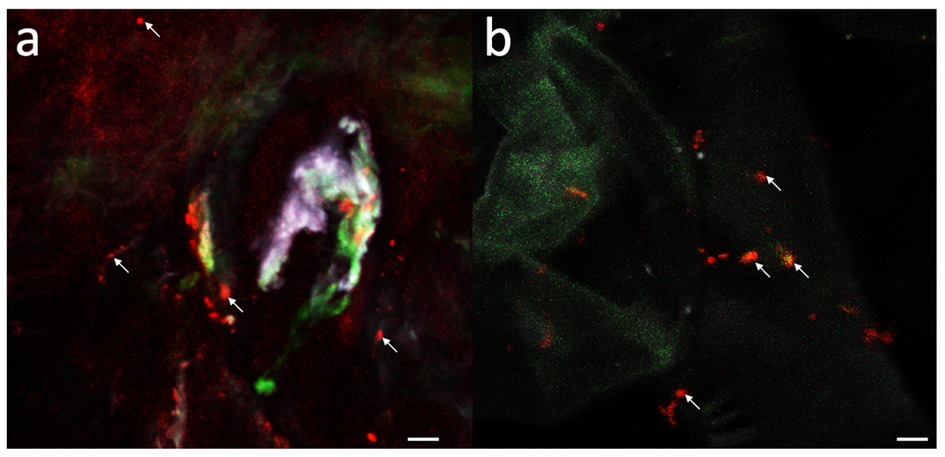
**

**Figure S6. Micrographs of bacterial colonization of fresh ripe strawberry tissues.** Bacteria visualization in the fruit pulp (a) and fruit surface (b). The white arrows in the micrographs indicate individual cells of the visualized bacteria colonizing fruit tissues. The scale bars on the micrographs indicate 10 μm.

**Table S1. Chemical characteristics of the soil on which the experiment was established.**

| **pH in H_2_O** | **Nog.** | **Cog.** | **P** | **K** | **Mg** | **Ca** | **Stot** |
| --- | --- | --- | --- | --- | --- | --- | --- |
|  | **%** | | **mg kg^-1^** | | | | |
| 7.55 | 0.29 | 3.72 | 2113 | 1848 | 6395 | 12860 | 522 |
| **P** | **K** | **B** | **Cu** | **Fe** | **Mn** | **Zn** | **Na** |
| **mg 100 g^-1^** | | **mg kg^-1^** | | | | | |
| 46.8 | 7.22 | 25.0 | 48.8 | 25420 | 718 | 109 | 206 |

**Table S2.** **Statistical tests on microbial abundance between strawberry compartments**

| **Bacterial community** | | | | | | | |
| --- | --- | --- | --- | --- | --- | --- | --- |
| **Compartment type: Kruskal-Wallis chi-squared = 56.561, df = 7, p-value = 0.00***** | | | | | | |  |
| **Cultivar: Kruskal-Wallis chi-squared = 0.46265, df = 1, p-value = 0.496** | | | | | |  |  |
| **Position (Belowground and phyllosphere): Kruskal-Wallis chi-squared = 43.882, df = 2, p-value = 0.00***** | | | | | | | |
|  | **Bulk soil** | **Rhizosphere** | **Leaves** | **Flowers** | **Immature fruits** | **Ripe fruits** | **Diseased fruits** |
| **Rhizosphere** | 0.883 |  |  |  |  |  |  |
| **Leaves** | 0.000*** | 0.000*** |  |  |  |  |  |
| **Flowers** | 0.001** | 0.000*** | 0.356 |  |  |  |  |
| **Immature fruits** | 0.000 | 0.000*** | 0.000*** | 0.008* |  |  |  |
| **Ripe fruits** | 0.000*** | 0.000*** | 0.977 | 0.478 | 0.001** |  |  |
| **Diseased fruits** | 0.000** | 0.000*** | 0.104 | 0.037* | 0.000*** | 0.116 |  |
| **Stored fruits** | 0.000** | 0.000*** | 0.008* | 0.001** | 0.000*** | 0.017* | 0.966 |
| **Fungal community** | | | | | | | |
| **Compartment type: Kruskal-Wallis chi-squared = 18.618, df = 7, p-value = 0.01** | | | | | | |  |
| **Cultivar: Kruskal-Wallis chi-squared = 9.5631, df = 1, p-value = 0.002** | | | | | | |  |
| **Position (Belowground and phyllosphere): Kruskal-Wallis chi-squared = 11.554, df = 1, p-value = 0.0007** | | | | | | | |
|  | **Bulk soil** | **Rhizosphere** | **Leaves** | **Flowers** | **Immature fruits** | **Ripe fruits** | **Diseased fruits** |
| **Rhizosphere** | 0.766 |  |  |  |  |  |  |
| **Leaves** | 0.017* | 0.017* |  |  |  |  |  |
| **Flowers** | 0.475 | 0.701 | 0.201 |  |  |  |  |
| **Immature fruits** | 0.017* | 0.017* | 0.766 | 0.160 |  |  |  |
| **Ripe fruits** | 0.017* | 0.088 | 0.701 | 0.475 | 0.533 |  |  |
| **Diseased fruits** | 0.766 | 0.766 | 0.543 | 1.000 | 0.701 | 0.701 |  |
| **Stored fruits** | 0.475 | 0.250 | 0.393 | 0.701 | 0.410 | 0.766 | 0.938 |

**Table S3: Alpha and Beta diversity indices for the bacterial community.**

| **Shannon diversity (Bacterial community)** | | | | | | |
| --- | --- | --- | --- | --- | --- | --- |
|  | **SS** | **df** | | **F** | **P** |  |
| All Compartments including bulk soil | 73.360 | 7 | | 29.070 | 0.000 | *** |
| Position: Belowground & Phyllosphere | 56.380 | 1 | | 100.600 | 0.000 | *** |
| **Exclude soil from the analysis (Two-way Anova)** | | | | | | |
| Compartment type | 45.580 | 6 | | 28.094 | 0.000 | *** |
| Host-cultivar | 2.300 | 1 | | 8.496 | 0.005 | ** |
| Compartment type: Cultivar | 6.810 | 6 | | 4.198 | 0.001 | ** |
| **Compartment-specific Shannon diversity differences between cultivars** | **SS** | **df** | | **F** | **P** |  |
| Diseased fruits | 3.765 | 1 | | 5.013 | 0.060 |  |
| Flowers | 0.010 | 1 | | 0.072 | 0.795 |  |
| Leaves | 4.374 | 1 | | 33.86 | 0.001 | *** |
| Ripe fruits | 0.022 | 1 | | 0.073 | 0.794 |  |
| Immature fruits | 0.505 | 1 | | 1.304 | 0.287 |  |
| Stored fruits | 0.194 | 1 | | 0.944 | 0.364 |  |
| Rhizosphere | 0.240 | 1 | | 3.765 | 0.081 |  |
| \| **Post-hoc analysis (Shannon diversity)** \| \| \| \| \| \| --- \| --- \| --- \| --- \| --- \| \| Compartment-specific comparison in bacterial diversity (Shannon) \| diff \| lwr \| upr \| p adj \| \| Rhizosphere & Bulk soil \| -0.778 \| -1.717 \| 0.161 \| 0.177 \| \| Leaves & Bulk soil \| -2.985 \| -3.975 \| -1.996 \| 0 \| \| Flowers & Bulk soil \| -2.917 \| -3.886 \| -1.947 \| 0 \| \| Immature fruits & Bulk soil \| -2.735 \| -3.705 \| -1.766 \| 0 \| \| Ripe fruits & Bulk soil \| -1.837 \| -2.790 \| -0.884 \| 0 \| \| Diseased fruits & Bulk soil \| -2.991 \| -3.980 \| -2.001 \| 0 \| \| Stored fruits & Bulk soil \| -1.900 \| -2.889 \| -0.909 \| 0 \| \| Leaves & Rhizosphere \| -2.2067 \| -3.035 \| -1.379 \| 0 \| \| Flowers & Rhizosphere \| -2.139 \| -2.943 \| -1.335 \| 0 \| \| Immature fruits & Rhizosphere \| -1.957 \| -2.761 \| -1.153 \| 0 \| \| Ripe fruits & Rhizosphere \| -1.058 \| -1.842 \| -0.275 \| 0.002 \| \| Diseased fruits & Rhizosphere \| -2.213 \| -3.040 \| -1.385 \| 0 \| \| Stored fruits & Rhizosphere \| -1.121 \| -1.949 \| -0.293 \| 0.002 \| \| Flowers & Leaves \| 0.068 \| -0.794 \| 0.931 \| 0.999 \| \| Immature fruits & Leaves \| 0.250 \| -0.613 \| 1.112 \| 0.985 \| \| Ripe fruits & Leaves \| 1.149 \| 0.305 \| 1.992 \| 0.001 \| \| Diseased fruits & Leaves \| -0.006 \| -0.891 \| 0.880 \| 1 \| \| Stored fruits & Leaves \| 1.086 \| 0.201 \| 1.971 \| 0.006 \| \| Immature fruits & Flowers \| 0.181 \| -0.658 \| 1.021 \| 0.998 \| \| Ripe fruits & Flowers \| 1.080 \| 0.260 \| 1.901 \| 0.003 \| \| Diseased fruits & Flowers \| -0.074 \| -0.937 \| 0.789 \| 0.999 \| \| Stored fruits & Flowers \| 1.018 \| 0.155 \| 1.881 \| 0.010 \| \| Ripe fruits & Immature fruits \| 0.899 \| 0.078 \| 1.719 \| 0.022 \| \| Diseased fruits & Immature fruits \| -0.255 \| -1.118 \| 0.607 \| 0.982 \| \| Stored fruits & Immature fruits \| 0.836 \| -0.026 \| 1.667 \| 0.064 \| \| Diseased fruits & Ripe fruits \| -1.154 \| -1.998 \| -0.310 \| 0.001 \| \| Stored fruits & Ripe fruits \| -0.062 \| -0.906 \| 0.782 \| 0.999 \| \| Stored fruits & Diseased fruits \| 1.092 \| 0.207 \| 1.977 \| 0.006 \| | | | | | | |
| **Observed ASVs (Bacterial community)** | | | | | | |
|  | **SS** | **df** | | **F** | **P** |  |
| All Compartments including bulk soil | 6.362 | 7 | | 18.790 | 0.000 | *** |
| Position: Belowground & Phyllosphere | 4.273 | 1 | | 58.790 | 0.000 | *** |
| **Exclude soil from the analysis (Two-way Anova)** | | | | | | |
| Compartment type | 4.492 | 6 | | 18.665 | 0.000 | *** |
| Host-cultivar | 0.298 | 1 | | 7.438 | 0.009 | ** |
| Compartment type: Cultivar | 0.710 | 6 | | 2.949 | 0.01 | * |
| **Compartment-specific richness (Observed ASVs) differences between cultivars** | **SS** | **df** | | **F** | **P** |  |
| Diseased fruits | 0.231 | 1 | | 2.446 | 0.162 |  |
| Flowers | 0.001 | 1 | | 0.024 | 0.88 |  |
| Leaves | 0.603 | 1 | | 13.73 | 0.008 | ** |
| Ripe fruits | 0.039 | 1 | | 1.369 | 0.272 |  |
| Immature fruits | 0.066 | 1 | | 1.891 | 0.206 |  |
| Stored fruits | 0.065 | 1 | | 1.912 | 0.209 |  |
| Rhizosphere | 0.003 | 1 | | 0.316 | 0.586 |  |
| \| **Post-hoc analysis (Observed ASVs)** \| \| \| \| \| \| --- \| --- \| --- \| --- \| --- \| \| Compartment-specific comparison in bacterial richness \| diff \| lwr \| upr \| pvalue \| \| Rhizosphere & Bulk soil \| -0.165 \| <0.00 \| 0.179 \| 0.804 \| \| Leaves & Bulk soil \| -0.858 \| <0.00 \| -0.495 \| 0.00 \| \| Flowers & Bulk soil \| -0.840 \| <0.00 \| -0.485 \| 0.00 \| \| Immature fruits & Bulk soil \| -0.706 \| <0.00 \| -0.351 \| 0.00 \| \| Ripe fruits & Bulk soil \| -0.405 \| <0.00 \| -0.056 \| 0.012 \| \| Diseased fruits & Bulk soil \| -0.790 \| <0.00 \| <0.00 \| 0.00 \| \| Stored fruits & Bulk soil \| -0.443 \| <0.00 \| <0.00 \| 0.007 \| \| Leaves & Rhizosphere \| -0.693 \| <0.00 \| <0.00 \| 0.00 \| \| Flowers & Rhizosphere \| -0.675 \| <0.00 \| <0.00 \| 0.00 \| \| Immature fruits & Rhizosphere \| -0.542 \| <0.00 \| <0.00 \| 0.00 \| \| Ripe fruits & Rhizosphere \| -0.240 \| <0.00 \| 0.047 \| 0.167 \| \| Diseased fruits & Rhizosphere \| -0.624 \| <0.00 \| -0.321 \| 0.00 \| \| Stored fruits & Rhizosphere \| -0.278 \| <0.00 \| 0.025 \| 0.096 \| \| Flowers & Leaves \| 0.0175 \| <0.00 \| 0.334 \| 1 \| \| Immature fruits & Leaves \| 0.152 \| <0.00 \| 0.468 \| 0.804 \| \| Ripe fruits & Leaves \| 0.452 \| 0 \| 0.761 \| 0.000 \| \| Diseased fruits & Leaves \| 0.068 \| <0.00 \| 0.393 \| 0.998 \| \| Stored fruits & Leaves \| 0.415 \| <0.00 \| 0.739 \| 0.004 \| \| Immature fruits & Flowers \| 0.134 \| <0.00 \| 0.442 \| 0.870 \| \| Ripe fruits & Flowers \| 0.435 \| 0.00 \| 0.735 \| 0.001 \| \| Diseased fruits & Flowers \| 0.051 \| <0.00 \| 0.367 \| 1 \| \| Stored fruits & Flowers \| 0.397 \| 0.00 \| 0.713 \| 0.005 \| \| Ripe fruits & Immature fruits \| 0.300 \| <0.00 \| 0.601 \| 0.050 \| \| Diseased fruits & Immature fruits \| -0.083 \| <0.00 \| 0.233 \| 0.991 \| \| Stored fruits & Immature fruits \| 0.263 \| <0.00 \| 0.579 \| 0.173 \| \| Diseased fruits & Ripe fruits \| -0.384 \| <0.00 \| -0.075 \| 0.006 \| \| Stored fruits & Ripe fruits \| -0.038 \| <0.00 \| 0.272 \| 1 \| \| Stored fruits & Diseased fruits \| 0.346 \| 0.00 \| 0.670 \| 0.028 \| | | | | | | |
| **Chao1 (Bacterial community)** | | | | | | |
|  | **SS** | **df** | | **F** | **P** |  |
| All Compartments including bulk soil | 7.437 | 7 | | 15.140 | 0.000 | *** |
| Position: Belowground & Phyllosphere | 4.59 | 1 | | 44.570 | 0.000 | *** |
| **Exclude soil from the analysis** | | | | | | |
| Compartment type | 5.479 | 6 | | 14.326 | 0.000 | *** |
| Host-cultivar | 0.400 | 1 | | 6.279 | 0.020 | * |
| Compartment type: Cultivar | 3.570 | 6 | | 1.863 | 0.1 |  |
| **Compartment-specific Chao1 diversity index differences between cultivars** | **SS** | **df** | | **F** | **P** |  |
| Diseased fruits | 0.175 | 1 | | 1.607 | 0.245 |  |
| Flowers | 0.001 | 1 | | 0.013 | 0.911 |  |
| Leaves | 0.664 | 1 | | 11.62 | 0.010 | * |
| Ripe fruits | 0.088 | 1 | | 1.653 | 0.231 |  |
| Immature fruits | 0.022 | 1 | | 0.314 | 0.591 |  |
| Stored fruits | 0.162 | 1 | | 3.527 | 0.102 |  |
| Rhizosphere | 0.001 | 1 | | 0.034 | 0.857 |  |
| \| **Post-hoc analysis (Chao1)** \| \| \| \| \| \| --- \| --- \| --- \| --- \| --- \| \| Compartment-specific comparison in bacterial richness (Chao1) \| diff \| lwr \| upr \| p \| \| Rhizosphere & Bulk soil \| -0.1601 \| -0.575 \| 0.254 \| 0.926 \| \| Leaves & Bulk soil \| -0.952 \| -1.390 \| -0.516 \| 0.00 \| \| Flowers & Bulk soil \| -0.884 \| -1.312 \| -0.456 \| 0.00 \| \| Immature fruits & Bulk soil \| -0.739 \| -1.167 \| -0.311 \| 0.00 \| \| Ripe fruits & Bulk soil \| -0.404 \| -0.824 \| 0.017 \| 0.069 \| \| Diseased fruits & Bulk soil \| -0.768 \| -1.205 \| -0.331 \| 0.00 \| \| Stored fruits & Bulk soil \| -0.398 \| -0.834 \| 0.039 \| 0.100 \| \| Leaves & Rhizosphere \| -0.793 \| -1.157 \| -0.427 \| 0.00 \| \| Flowers & Rhizosphere \| -0.723 \| -1.078 \| -0.369 \| 0.00 \| \| Immature fruits & Rhizosphere \| -0.579 \| -0.933 \| -0.223 \| 0.00 \| \| Ripe fruits & Rhizosphere \| -0.243 \| -0.589 \| 0.103 \| 0.365 \| \| Diseased fruits & Rhizosphere \| -0.608 \| -0.973 \| -0.242 \| 0.00 \| \| Stored fruits & Rhizosphere \| -0.237 \| -0.602 \| 0.128 \| 0.471 \| \| Flowers & Leaves \| 0.069 \| -0.312 \| 0.449 \| 0.999 \| \| Immature fruits & Leaves \| 0.213 \| -0.167 \| 0.594 \| 0.653 \| \| Ripe fruits & Leaves \| 0.547 \| 0.1763 \| 0.921 \| 0.000 \| \| Diseased fruits & Leaves \| 0.184 \| -0.206 \| 0.575 \| 0.817 \| \| Stored fruits & Leaves \| 0.555 \| 0.164 \| 0.945 \| 0.001 \| \| Immature fruits & Flowers \| 0.145 \| -0.226 \| 0.515 \| 0.923 \| \| Ripe fruits & Flowers \| 0.480 \| 0.118 \| 0.842 \| 0.002 \| \| Diseased fruits & Flowers \| 0.116 \| -0.265 \| 0.496 \| 0.979 \| \| Stored fruits & Flowers \| 0.486 \| 0.106 \| 0.867 \| 0.004 \| \| Ripe fruits & Immature fruits \| 0.335 \| -0.027 \| 0.697 \| 0.089 \| \| Diseased fruits & Immature fruits \| -0.029 \| -0.410 \| 0.352 \| 1 \| \| Stored fruits & Immature fruits \| 0.342 \| -0.039 \| 0.722 \| 0.111 \| \| Diseased fruits & Ripe fruits \| -0.364 \| -0.737 \| 0.008 \| 0.059 \| \| Stored fruits & Ripe fruits \| 0.006 \| -0.366 \| 0.379 \| 1 \| \| Stored fruits & Diseased fruits \| 0.371 \| -0.019 \| 0.761 \| 0.075 \| | | | | | | |
| **Bacterial community β-diversity Analysis (PERMANOVA)** | | | | | | |
|  | df | Sums of Sqs | | F.Model | % variation | P value |
| All Compartments including bulk soil | 7 | 12.196 | | 7.961 | 45.040 | 0.001*** |
| Position: Belowground & Phyllosphere | 1 | 5.376 | | 18.332 | 19.900 | 0.001*** |
| **Exclude soil from the analysis to investigate cultivar effect** | | | | | | |
| Compartment type | 6 | 9.895 | | 8.589 | 41.400 | 0.001*** |
| Host-Cultivar | 1 | 0.399 | | 2.078 | 1.669 | 0.017* |
| Plant organ: Cultivar | 6 | 2.854 | | 2.478 | 11.943 | 0.001*** |
| **Compartment-specific Beta diversity comparison (PERMANOVA Adonis)** | F.Model | | % variation | p.value | p.adjusted |  |
| Diseased fruits & Flowers | 5.161 | 23.3 | | 0.001 | 0.021 | * |
| Diseased fruits & Leaves | 6.819 | 29.9 | | 0.001 | 0.021 | * |
| Diseased fruits & Stored fruits | 5.683 | 26.2 | | 0.001 | 0.021 | * |
| Diseased fruits & Ripe fruits | 8.008 | 30.8 | | 0.001 | 0.021 | * |
| Diseased fruits & Rhizosphere | 12.662 | 40.0 | | 0.001 | 0.021 | * |
| Diseased fruits & Immature fruits | 5.514 | 24.5 | | 0.001 | 0.021 | * |
| Flowers & Leaves | 2.799 | 14.1 | | 0.001 | 0.021 | * |
| Flowers & Stored fruits | 3.539 | 17.2 | | 0.001 | 0.021 | * |
| Flowers & Ripe fruits | 3.246 | 14.6 | | 0.001 | 0.021 | * |
| Flowers & Rhizosphere | 14.028 | 41.2 | | 0.001 | 0.021 | * |
| Flowers & Immature fruits | 1.851 | 9.3 | | 0.02 | 0.42 |  |
| Leaves & Stored fruits | 5.056 | 24.0 | | 0.001 | 0.021 | * |
| Leaves & Ripe fruits | 2.995 | 14.3 | | 0.001 | 0.021 | * |
| Leaves & Rhizosphere | 17.033 | 47.3 | | 0.001 | 0.021 | * |
| Leaves & Immature fruits | 2.542 | 13.0 | | 0.004 | 0.084 |  |
| Stored fruits & Ripe fruits | 4.429 | 19.7 | | 0.001 | 0.021 | * |
| Stored fruits & Rhizosphere | 18.420 | 49.2 | | 0.001 | 0.021 | * |
| Stored fruits & Immature fruits | 3.574 | 17.4 | | 0.001 | 0.021 | * |
| Ripe fruits & Rhizosphere | 20.288 | 49.1 | | 0.001 | 0.021 | * |
| Ripe fruits & Immature fruits | 1.663 | 8.0 | | 0.035 | 0.735 |  |
| Rhizosphere & Immature fruits | 15.053 | 42.9 | | 0.001 | 0.021 | * |
| **Beta diversity due to cultivar influence in the different strawberry compartments (PERMANOVA)** | | | | | | |
|  | df | Sums of Sqs | | F. Model | % variation | P value |
| Diseased fruits | 1 | 0.712 | | 2.87 | 29.0 | 0.046 |
| Flowers | 1 | 0.664 | | 2.793 | 25.8 | 0.01* |
| Leaves | 1 | 0.627 | | 4.019 | 36.5 | 0.062 |
| Ripe fruits | 1 | 0.340 | | 2.257 | 20.0 | 0.007** |
| Immature fruits | 1 | 0.036 | | 1.466 | 16.8 | 0.051 |
| Stored fruits | 1 | 0.286 | | 1.735 | 19.8 | 0.046 |
| Rhizosphere | 1 | 0.235 | | 1.478 | 12.8 | 0.062 |

Significant differences are marked by asterisks as follows: 0.00***; 0.001 **; 0.01 *; 0.05 . ; and 0.1 ' ' 1

**Table S4: A statistical summary of alpha and beta diversity indices for the fungal community.**

| **Fungal richness (Observed ASVs)** | | | | | | | |
| --- | --- | --- | --- | --- | --- | --- | --- |
| **Position (Belowground & Phyllosphere): Kruskal-Wallis chi-squared = 29.628, df = 1, p-value = 0.00***** | | | | | | |  |
| **Compartment type: Kruskal-Wallis chi-squared = 47.708, df = 7, p-value 0.00***** | | | | | |  |  |
| **Cultivar influence (richness): Kruskal-Wallis chi-squared = 10.688, df = 1, p-value = 0.001*** | | | | | | | |
| Compartment | **Bulk soil** | **Rhizosphere** | **Leaves** | **Flowers** | **Immature fruits** | **Ripe fruits** | **Diseased fruits** |
| Rhizosphere | 0.123 |  |  |  |  |  |  |
| Leaves | 0.005* | 0.058* |  |  |  |  |  |
| Flowers | 0.004** | 0.004** | 0.038* |  |  |  |  |
| Immature fruits | 0.014* | 0.006* | 0.008* | 0.072* |  |  |  |
| Ripe fruits | 0.003** | 0.004** | 0.032* | 0.975 | 0.124 |  |  |
| Diseased fruits | 0.006* | 0.004** | 0.0023* | 0.012* | 0.138 | 0.012* |  |
| Stored fruits | 0.004** | 0.004** | 0.006* | 0.450 | 0.127 | 0.450 | 0.039^*^ |
| **Shannon diversity index (Fungal community)** | | | | | | | |
| **Position (Belowground & Phyllosphere): Kruskal-Wallis chi-squared = 33.066, df = 1, p-value = 8.908e-09** | | | | | | |  |
| **Compartment type: Kruskal-Wallis chi-squared = 56.531, df = 7, p-value = 7.407e-10** | | | | | |  |  |
| **Cultivar influence (Shannon): Kruskal-Wallis chi-squared = 8.9144, df = 1, p-value = 0.002829** | | | | | | |  |
| Compartment | Bulk soil | Rhizosphere | Leaves | Flowers | Immature fruits | Ripe fruits | Diseased fruits |
| Rhizosphere | 0.057 |  |  |  |  |  |  |
| Leaves | 0.000** | 0.044** |  |  |  |  |  |
| Flowers | 0.000** | 0.000** | 0.000* |  |  |  |  |
| Immature fruits | 0.007* | 0.001** | 0.001** | 0.210 |  |  |  |
| Ripe fruits | 0.000** | 0.000** | 0.000** | 0.466 | 0.466 |  |  |
| Diseased fruits | 0.001* | 0.000** | 0.000** | 0.060 | 0.208 | 0.097 |  |
| Stored fruits | 0.000** | 0.000** | 0.000** | 0.121 | 0.898 | 0.311 | 0.099 |
| **Chao1 (Fungal community)** | | | | | | | |
| **Position (Belowground & Phyllosphere): Kruskal-Wallis chi-squared = 27.488, df = 1, p-value = 0.00***** | | | | | | |  |
| **Compartment type: Kruskal-Wallis chi-squared = 41.573, df = 7, p-value = 0.00*****  **Cultivar influence (Chao1): Kruskal-Wallis chi-squared = 11.047, df = 1, p-value = 0.001**** | | | | | |  |  |
| Compartment | Bulk soil | Rhizosphere | Leaves | Flowers | Immature fruits | Ripe fruits | Diseased fruits |
| Rhizosphere | 0.1095 |  |  |  |  |  |  |
| Leaves | 0.0024** | 0.0647 |  |  |  |  |  |
| Flowers | 0.002** | 0.004** | 0.065 |  |  |  |  |
| Immature fruits | 0.009* | 0.006* | 0.018* | 0.285 |  |  |  |
| Ripe fruits | 0.002** | 0.008* | 0.234 | 0.833 | 0.312 |  |  |
| Diseased fruits | 0.007* | 0.004** | 0.009* | 0.018* | 0.123 | 0.016* |  |
| Stored fruits | 0.002** | 0.002** | 0.009* | 0.680 | 0.471 | 0.312 | 0.039* |
| \| **Compartment-specific differences in fungal alpha diversity between cultivars (Kruskal-Wallis chi-square test and Wilcoxon Rank Sum Tests)** \| \| \| \| \| --- \| --- \| --- \| --- \| \| **Compartment-specific differences in fungal alpha diversity between cultivars** \| **pvalues (White ananas Vs Mara de Bois)** \| \| \| \| Observed ASVs \| Shannon \| Chao1 \| \| Rhizosphere \| 0.227 \| 0.187 \| 0.266 \| \| Leaves \| 0.911 \| 1 \| 0.992 \| \| Flowers \| 0.563 \| 0.014* \| 0.843 \| \| Immature fruits \| 0.841 \| 0.867 \| 1 \| \| Ripe fruits \| 0.038* \| 0.581 \| 0.038 \| \| Diseased fruits \| 0.132 \| 0.114 \| 0.185 \| \| Stored fruits \| 0.495 \| 0.639 \| 0.744 \| | | | | | |  | |
| **β-diversity in the Fungal community (PERMANOVA)** | | | | | |  | |
|  | df | Sums of Sqs | F. Model | % variation | P value |  |  |
| All Compartments including bulk soil | 7 | 12.577 | 14.748 | 61.0 | 0.001*** |  |  |
| Position: Belowground & Phyllosphere | 1 | 6.258 | 31.026 | 30.4 | 0.001*** |  |  |
| **Exclude soil from the analysis to investigate cultivar effect** | | | | | |  |  |
| Compartment type | 6 | 10.118 | 21.226 | 59.54 | 0.001*** |  |  |
| Cultivar | 1 | 0.384 | 4.827 | 2.26 | 0.001** |  |  |
| Compartment type: Cultivar | 6 | 2.201 | 4.617 | 13.0 | 0.001*** |  |  |
| **Pairwise comparison between compartment-specific microbial Beta Diversity (PERMANOVA adonis)** | | | | | |  |  |
| Compartment | F. Model | R2 | p.value | p. adjusted |  |  |  |
| Diseased fruits & Flowers | 19.7822 | 53.8 | 0.001 | 0.021* |  |  |  |
| Diseased fruits & Leaves | 19.9761 | 52.6 | 0.001 | 0.021* |  |  |  |
| Diseased fruits & Stored fruits | 24.1614 | 61.7 | 0.001 | 0.021* |  |  |  |
| Diseased fruits & Ripe fruits | 30.7998 | 63.1 | 0.001 | 0.021* |  |  |  |
| Diseased fruits & Rhizosphere | 9.6582 | 36.2 | 0.001 | 0.021* |  |  |  |
| Diseased fruits & Immature fruits | 13.7552 | 55.5 | 0.007 | 0.147 |  |  |  |
| Flowers & Leaves | 6.9975 | 25 | 0.001 | 0.021* |  |  |  |
| Flowers & Stored fruits | 3.5484 | 16.5 | 0.032 | 0.672 |  |  |  |
| Flowers & Ripe fruits | 1.7715 | 7.8 | 0.133 | 1 |  |  |  |
| Flowers & Rhizosphere | 17.2584 | 46.3 | 0.001 | 0.021* |  |  |  |
| Flowers & Immature fruits | 0.6976 | 4.7 | 0.503 | 1 |  |  |  |
| Leaves & Stored fruits | 17.0198 | 47.3 | 0.001 | 0.021* |  |  |  |
| Leaves & Ripe fruits | 11.9251 | 35.2 | 0.001 | 0.021* |  |  |  |
| Leaves & Rhizosphere | 16.1594 | 43.5 | 0.001 | 0.021* |  |  |  |
| Leaves & Immature fruits | 4.6572 | 23.7 | 0.003 | 0.063 |  |  |  |
| Stored fruits & Ripe fruits | 10.8819 | 36.4 | 0.001 | 0.021* |  |  |  |
| Stored fruits & Rhizosphere | 18.5351 | 50.7 | 0.001 | 0.021* |  |  |  |
| Stored fruits & Immature fruits | 9.8174 | 45 | 0.002 | 0.042* |  |  |  |
| Ripe fruits & Rhizosphere | 23.9633 | 53.3 | 0.001 | 0.021* |  |  |  |
| Ripe fruits & Immature fruits | 1.6314 | 9.8 | 0.145 | 1 |  |  |  |
| Rhizosphere & Immature fruits | 10.3062 | 42.4 | 0.001 | 0.021* |  |  |  |
| Phyllosphere & Below ground | 25.9265 | 28.2 | 0.001 | 0.001** |  |  |  |
| **Beta diversity due to cultivar influence in different the strawberry compartments (PERMANOVA)** | | | | | |  |  |
| **Compartment** | df | Sums of Sqs | F. Model | % variation | P value |  |  |
| Diseased fruits | 1 | 1.208 | 61.11 | 91.1 | 0.036* |  |  |
| Flowers | 1 | 0.374 | 7.557 | 45.6 | 0.012* |  |  |
| Leaves | 1 | 0.406 | 5.229 | 34.3 | 0.003** |  |  |
| Ripe fruits | 1 | 0.058 | 2.878 | 22.3 | 0.009** |  |  |
| Immature fruits | 1 | 0.036 | 1.466 | 32.8 | 0.4 |  |  |
| Stored fruits | 1 | 0.075 | 5.606 | 44.5 | 0.018 * |  |  |
| Rhizosphere | 1 | 0.428 | 1.493 | 14.2 | 0.031* |  |  |

**Significant differences are marked by asterisks as follows: 0.00***; 0.001 **; 0.01 *; 0.05 . ; and 0.1 ' ' 1**

**Table S5. Proportion of taxa transferred from soil (source) to aboveground parts (sink).**

| **Cultivar** | **Source-Sink** | **Bacteria [%]** | **Fungi [%]** |
| --- | --- | --- | --- |
| Cultivar 1 | Bulk soil-Immature fruits | 1 | 0 |
| Cultivar 2 | Bulk soil-Immature fruits | 0 | 0 |
| Cultivar 1 | Bulk soil-Ripe fruits | 1 | 0 |
| Cultivar 2 | Bulk soil-Ripe fruits | 7 | 0 |
| Cultivar 1 | Bulk soil-Flowers | 0 | 0 |
| Cultivar 2 | Bulk soil-Flowers | 0 | 0 |
| Cultivar 1 | Bulk soil-Leaves | 0 | 0 |
| Cultivar 2 | Bulk soil-Leaves | 1 | 1 |

**Table S6: A statistical summary of alpha and beta diversity indices for the fungal community**

| **β-diversity due to cultivar and fruit condition** | | | | | | | | | | |
| --- | --- | --- | --- | --- | --- | --- | --- | --- | --- | --- |
| **Bacterial community** | | | | | | **Fungal community** | | | | |
|  | Df | Sums of Sqs | F. Model | R2 | Pr(>F) | Df | Sums of Sqs | F. Model | R2 | Pr(>F) |
| Fruit condition | 2 | 2.694 | 7.438 | 33.0 | 0.001*** | 2 | 3.466 | 96.387 | 66.4 | 0.001*** |
| Cultivar | 1 | 0.437 | 2.410 | 5.3 | 0.006** | 1 | 0.389 | 21.653 | 7.6 | 0.001*** |
| Fruit condition: Cultivar | 2 | 0.879 | 2.428 | 11 | 0.001*** | 2 | 0.952 | 26.466 | 18.2 | 0.001*** |

Significant differences are marked by asterisks as follows: 0.00***; 0.001 **; 0.01 *; 0.05 . ; and 0.1 ' ' 1
